# The potential role of early life feeding patterns in shaping the infant fecal metabolome: implications for neurodevelopmental outcomes

**DOI:** 10.1038/s44324-023-00001-2

**Published:** 2023-12-13

**Authors:** Bridget Chalifour, Elizabeth A. Holzhausen, Joseph J. Lim, Emily N. Yeo, Natalie Shen, Dean P. Jones, Bradley S. Peterson, Michael I. Goran, Donghai Liang, Tanya L. Alderete

**Affiliations:** 1 Department of Integrative Physiology, University of Colorado Boulder, Boulder, CO, USA.; 2 Rollins School of Public Health, Emory University, Atlanta, GA, USA.; 3 School of Medicine, Emory University, Atlanta, GA, USA.; 4 Children’s Hospital Los Angeles, Los Angeles, CA, USA.

## Abstract

Infant fecal metabolomics can provide valuable insights into the associations of nutrition, dietary patterns, and health outcomes in early life. Breastmilk is typically classified as the best source of nutrition for nearly all infants. However, exclusive breastfeeding may not always be possible for all infants. This study aimed to characterize associations between levels of mixed breastfeeding and formula feeding, along with solid food consumption and the infant fecal metabolome at 1- and 6-months of age. As a secondary aim, we examined how feeding-associated metabolites may be associated with early life neurodevelopmental outcomes. Fecal samples were collected at 1- and 6-months, and metabolic features were assessed via untargeted liquid chromatography/high-resolution mass spectrometry. Feeding groups were defined at 1-month as 1) exclusively breastfed, 2) breastfed >50% of feedings, or 3) formula fed ≥50% of feedings. Six-month groups were defined as majority breastmilk (>50%) or majority formula fed (≥50%) complemented by solid foods. Neurodevelopmental outcomes were assessed using the Bayley Scales of Infant Development at 2 years. Changes in the infant fecal metabolome were associated with feeding patterns at 1- and 6-months. Feeding patterns were associated with the intensities of a total of 57 fecal metabolites at 1-month and 25 metabolites at 6-months, which were either associated with increased breastmilk or increased formula feeding. Most breastmilk-associated metabolites, which are involved in lipid metabolism and cellular processes like cell signaling, were associated with higher neurodevelopmental scores, while formula-associated metabolites were associated with lower neurodevelopmental scores. These findings offer preliminary evidence that feeding patterns are associated with altered infant fecal metabolomes, which may be associated with cognitive development later in life.

## INTRODUCTION

Breastmilk is considered an ideal source of nutrition. Benefits of breastfeeding include protection against allergies^1^, better immune system development^2^, lower risk of childhood obesity^3,4^, and optimal brain development^5^. For these reasons, the World Health Organization and the United Nations International Children’s Emergency Fund recommend exclusive breastfeeding for the first six months of life^6^. Despite this recommendation, exclusive breastfeeding may not always be possible or suitable for all mothers and infants. In the United States, only 62.6% of infants are exclusively breastfed immediately following birth. This rate drops to 24.9% by 6-months of age^7^. While infant formula is designed to provide all the necessary nutrients for infant growth and development, it has been linked with infant hospitalizations and infections^8^, childhood obesity^9^, and lowered levels of docosahexaenoic acid (DHA), an important fatty acid related to brain development^10,11^.

Breastmilk is hypothesized to improve infant health in part through beneficial impacts on the developing gut microbiome and fecal metabolome^12–16^. Around 6-months of age, infants begin to consume small amounts of complementary solid foods, which can further impact the gut microbiome and fecal metabolome^17–19^. The fecal metabolome can serve as a functional readout of gut bacteria^20^, which can be dynamically impacted by early life feeding patterns, and it can influence infant health through the diffusion of metabolites into circulation^21–23^. Thus, characterizing the infant fecal metabolome and the individual metabolites within may provide important mechanistic insights regarding the association of early-life nutrition with developmental outcomes later in life^5,24–30^. For example, growing evidence suggests that the infant gut microbiome^31–33^, fecal metabolome^34,35^, and prolonged breastfeeding^36,37^ are each associated with improved neurodevelopmental outcomes. This is important because the brain grows to 80–90% of its adult volume in the first two years of life, establishing the structural foundations of cognitive and motor development^38–40^. Prior studies have nevertheless not examined how dietary patterns are associated with characteristics of the infant fecal metabolome and brain development in early life.

Previous studies have shown that the infant fecal metabolome changes based on infant age^41^, delivery mode^42^, and antibiotic usage^43,44^. The fecal metabolome also clearly depends on whether infants are exclusively breastfed or exclusively formula fed^45–48^. As complementary solid foods are introduced, fecal metabolomic profiles begin to converge and the metabolome of breastfed infants is indistinguishable from that of formula fed infants by 1 year of age^46,47^. Yet, no studies have examined how levels of mixed feeding, which is a far more common feeding choice, impact the infant fecal metabolome in the first 6 months of life. Therefore, we sought to determine if mixed feeding was associated with the alterations in the infant fecal metabolome in 112 Latino infants from the Southern California Mother’s Milk Study. Our primary aim was to determine if infant feeding patterns, including varying proportions of breastmilk or formula, were associated with alterations in the infant fecal metabolome at 1- and 6-months of age. At 6-months of age, we then sought to determine whether the infant fecal metabolome differed among infants who received solid foods in addition to either breastmilk or formula. As a secondary aim, we explored whether feeding-associated fecal metabolites were associated with neurodevelopmental outcomes at 2 years of age.

## RESULTS

### Study population characteristics

This study examined 112 Latino mother-infant pairs; general population characteristics are shown in Table 1. At the 1-month postpartum visit, average maternal age was 29.0 ± 6.3 years old (18–35), and average maternal pre-pregnancy body mass index was 28.5 ± 5.8 kg/m^2^. Most families were of a lower socioeconomic status (SES), with an average Hollingshead Index of 26.5 ± 12.0. Average infant age in days at each fecal metabolome assessment was 32.5 ± 3.4 days at 1-month and 185.9 ± 8.1 days at 6-months. Roughly half of the infants were female (53.6%), most were born vaginally (72.3%), and 10.7% received antibiotics, based on self-reported questionnaires. The average age of introduction to solid foods was 5.9 ± 1.7 months for all infants (Supplementary Table 1) and 5.4 ± 0.7 months for only infants included in 6-month analyses.

### The infant fecal metabolome is associated with infant feeding patterns

**Fig. 1:**
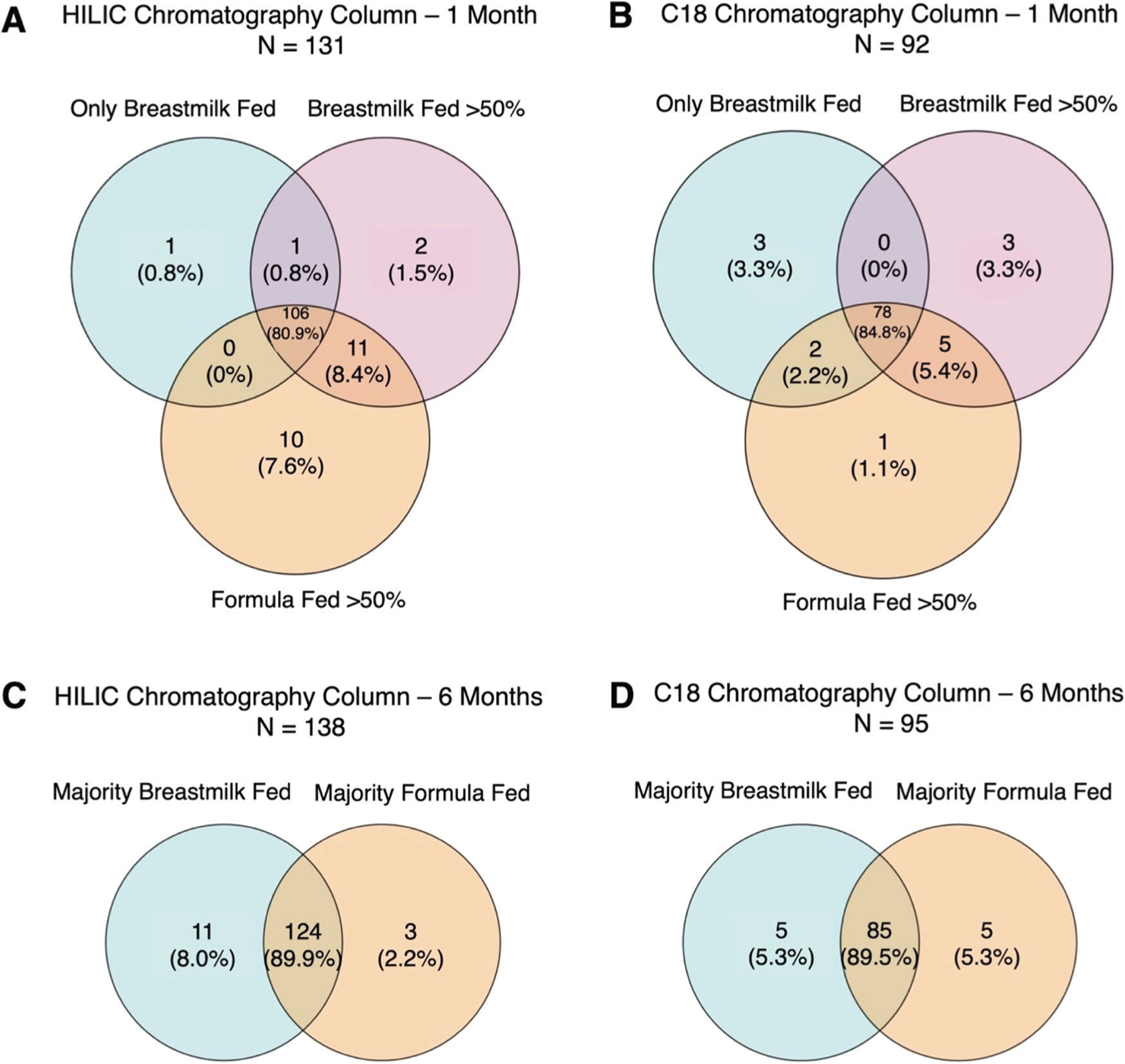
Confirmed metabolites observed across feeding groups in at least 50% of samples at 1- and 6-months in the HILIC and C18 chromatography columns. Confirmed metabolites observed in at least 50% of samples across feeding groups at 1-month of age in the HILIC (**A**) and C18 (**B**) chromatography columns and at 6-months of age in the HILIC (**C**) and C18 (**D**) chromatography columns.

Overall, we were able to confirm the chemical identities of 143 unique metabolites from HILIC chromatography and 104 metabolites from C18 chromatography with Level 1 evidence (i.e., features whose m/z and retention time could be matched to authentic standards with MS/MS under identical conditions). Among these confirmed metabolites, many were conserved across feeding groups at 1-month (exclusively breastfed for 100% of feedings, breastfed >50% of feedings, or formula fed ≥50% of feedings) and 6-months (majority breastfed with complementary solid foods). For example, 106/143 metabolites in the HILIC column were observed in at least 50% of samples within each feeding group, as were 78/104 metabolites in the C18 column at 1-month (Fig. 1). Similarly, 124/143 metabolites in the HILIC column were observed in at least 50% of samples within each feeding group, as were 85/104 metabolites in the C18 column at 6-months.

**Fig. 2:**
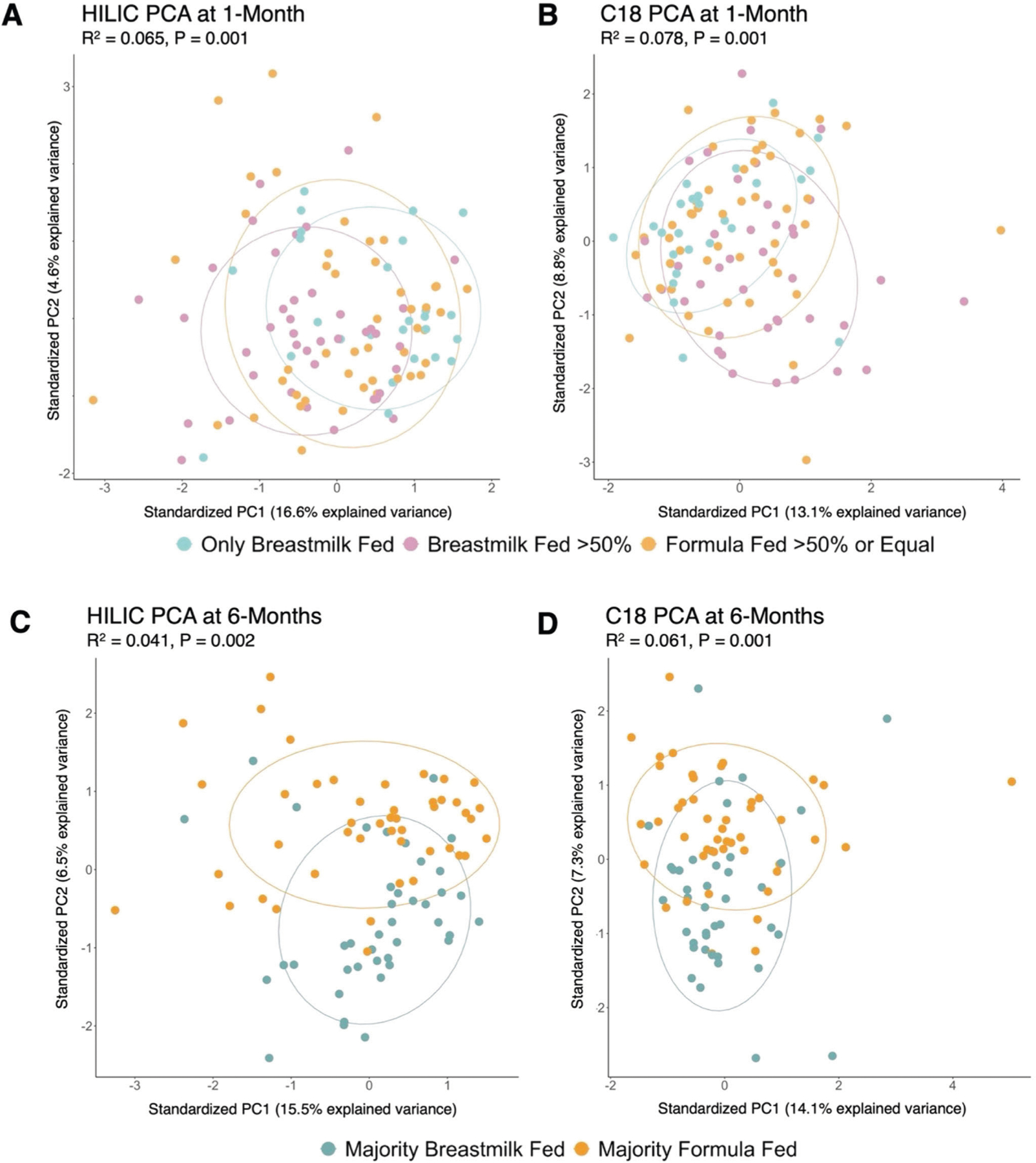
Ordination plot of principal components 1 and 2 for confirmed metabolites detected in HILIC and C18 chromatography columns by feeding group at 1- and 6-months of age. Principal component (PC) analysis of fecal metabolomic data from infants at 1- (top) and 6-months (bottom) of age. The plots show the first two PCs, which explain a total of 21.2% of the variance in the data in HILIC 1-month samples (**A**), 21.9% in C18 1-month samples (**B**), 22.0% in HILIC 6-month samples (**C**), and 21.4% in C18 6-month samples (**D**). The left plots show results from the HILIC chromatography column, and the right plots show the C18 chromatography column. Points are colored by infant feeding group, with corresponding legends to each timepoint denoted below. R^2^ and *P* values calculated using Permutational Multivariate Analysis of Variance (PERMANOVA), with infant feeding group as the explanatory variable. Plots show that infant fecal metabolome compositions at 1- and 6-months are significantly influenced by infant feeding patterns.

As the presence and intensities of confirmed metabolites appeared to change across feeding groups (Figs. 1, 2), we performed non-parametric univariate permutational multivariate analysis of variance tests (PERMANOVA) to determine if this variability could be attributed to feeding groups. We found that metabolomic variation was indeed driven by feeding group at both 1- and 6-months. At 1-month, 6.5% (*P* = 0.001) of the variability in confirmed metabolites in the HILIC column and 7.8% (*P* = 0.001) of the variability in confirmed metabolites in the C18 column could be attributed to infant feeding grouping. At 6-months, 4.1% (*P* = 0.002) of the variability in confirmed metabolites in the HILIC column and 6.1% (*P* = 0.001) of the variability in confirmed metabolites in the C18 column could be attributed to infant feeding grouping.

### Infant fecal metabolites are associated with increased breastmilk or formula-feeding

We examined the associations of metabolite intensities with feeding groups using linear models that adjusted for infant age in days and mode of delivery. At 1-month, there were 35 metabolites significantly associated with infant feeding in the HILIC chromatography column and 22 metabolites in the C18 chromatography column (*P*_BH_ < 0.05). Of these, the intensity of 17 metabolites (e.g., kynurenine, cholesterol, heptadecanoate, eicosadienoic acid) were positively associated with increased proportion of breastmilk feedings (the top ten most significantly associated metabolites are shown in Tables 2, 3; all significant metabolite results are presented in Supplementary File 1). Additionally, 40 metabolites (e.g., glyceric acid, thymidine, proline, laurate, myristate, and hypoxanthine) were positively associated with increased proportion of formula feedings. At 1-month of age, variations in metabolite intensities by feeding group were consistently monotonic as intensities either consistently increased or decreased as the proportion of breastmilk or formula feedings increased (Supplementary Figs. 1–4).

At 6-months, there were nine metabolites significantly associated with infant feeding in the HILIC chromatography column and 16 metabolites in the C18 chromatography column (*P*_BH_ < 0.05). Tables 2 and 3 summarize the top 10 most significant results for each chromatography column. The intensity of 13 metabolites (e.g., kynurenine, cholesterol, methyl vanillate/homovanillate, 6-deoxy-galactose (fucose)/rhamnose) were positively associated with increased proportion of breastmilk feedings. At the same time, increased proportion of formula feedings was associated with higher intensities of 12 other metabolites (e.g., those associated with amino acid metabolism like glyceric acid, glycerate, and lysine, and nucleotide metabolism like thymidine and hypoxanthine).

### Infant fecal metabolites associated with feeding are also associated with neuro-developmental outcomes at two years

**Fig. 3:**
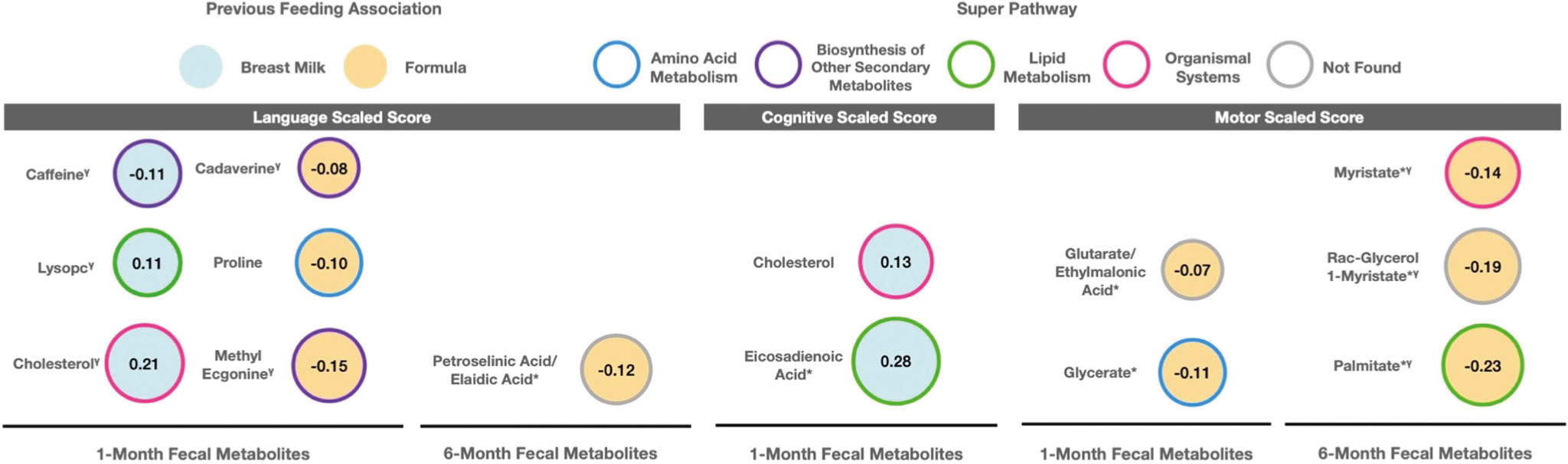
Metabolites that were significantly associated with feeding patterns at 1- and 6-months detected by HILIC and C18 chromatography columns that were also significantly associated with Bayley Scores at 2 years. Metabolites that were significantly associated with feeding patterns at 1- and 6-months detected by HILIC and C18 chromatography columns that were also significantly associated with Bayley Scores at 2 years. Results are based multivariate linear models that adjusted for infant birth weight and mode of delivery. Results from these models were adjusted using the Benjamini-Hochberg (BH) procedure within each neurodevelopmental score. An asterisk (*) next to a metabolite name indicates that it was a C18 metabolite, no (*) indicates HILIC. Outline border indicates the super pathway associated with each metabolite. All findings were statistically significant at unadjusted *P* < 0.05 except for those indicated (*γ* = *P*_BH_ ≤ 0.20).

As a secondary aim, we sought to determine if feeding-associated fecal metabolites were also associated with neurodevelopmental outcomes at 2 years of age. To accomplish this, we examined associations between metabolites found to be significantly associated with feeding patterns and neurodevelopmental outcomes (Bayley cognitive, motor, and language scaled scores) at 2 years of age. Results from this analysis broadly indicated that metabolites found to be positively associated with breastfeeding were also associated with higher Bayley scores, while metabolites found to be positively associated with formula feeding were also associated with lower Bayley scores at 2 years of age (Fig. 3). While some metabolites were very close to our multiple testing threshold of *P*_BH_ ≤ 0.20, others exceeded this threshold and did not hold up to multiple correction testing. However, we felt it was important to report all significant metabolites at the uncorrected *P* value level, as there were consistent neurodevelopmental patterns based on feeding group. The only metabolite to break these trends was caffeine, which was found to be positively associated with breastfeeding at 1-month but was negatively associated with language scaled score (*β* = −0.11; *P* = 0.03; *P*_BH_ = 0.21). We observed positive associations between lysoPC(16:0) (*β* = 0.11; *P* = 0.02; *P*_BH_ = 0.21) and cholesterol (*β* = 0.21; *P* = 0.02; *P*_BH_ = 0.21) with language scaled scores at 2 years—importantly, our analysis also revealed that these metabolites were positively associated with more breastfeeding. Cholesterol and eicosadienoic acid were also positively associated with breastfeeding and we found that these two metabolites were associated with higher cognitive scaled scores (*β* = 0.13; *P* = 0.04; *P*_BH_ = 0.86; *β* = 0.28; *P* = 0.03; *P*_BH_ = 0.65; respectively).

**Fig. 4:**
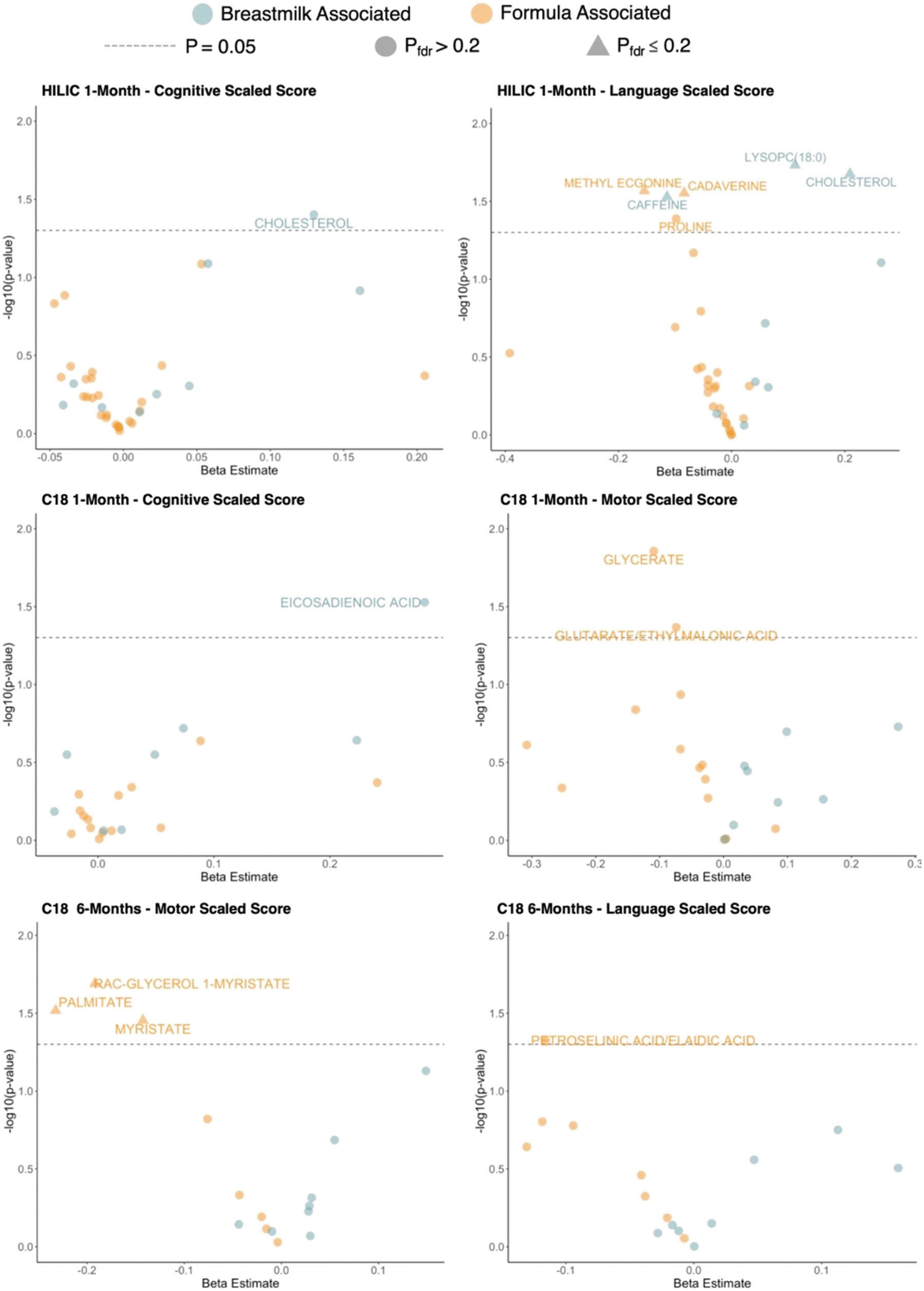
Summary of the associations between HILIC and C18 1- and 6-month metabolites that were significantly associated with infant feeding group and Bayley Scores at 2 years. Estimates were generated using linear models that adjusted for infant birth weight and mode of delivery. *P* values were adjusted for multiple testing using the Benjamini-Hochberg (BH) procedure. The dashed gray line corresponds to *P* = 0.05. Points are colored by previous feeding association (orange or blue), and triangular points indicate *P*_BH_ < 0.2, while circular points indicate *P*_BH_ > 0.2.

We also observed negative associations between several fecal metabolites and neurodevelopmental outcomes previously found to be positively associated with formula feeding. These metabolites included cadaverine (*β* = −0.08; *P* = 0.03; *P*_BH_ = 0.21), proline (*β* = −0.10; *P* = 0.04; *P*_BH_ = 0.24), and methyl ecgonine (*β* = −0.15; *P* = 0.03; *P*_BH_ = 0.21) with language scaled scores at 2 years. The metabolites glycerate and glutarate/ethylmalonic acid were also positively associated with formula feedings and negatively associated with motor scaled scores (*β* = −0.11; *P* = 0.01; *P*_BH_ = 0.31; *β* = −0.07; *P* = 0.04; *P*_BH_ = 0.47; respectively). Likewise, metabolites significantly associated with majority formula feeding at 6-months showed significant negative associations with Bayley scores at 2 years (Fig. 3). Myristate (*β* = −0.14; *P* = 0.04; *P*_BH_ = 0.19), rac-glycerol 1-myristate (*β* = −0.19; *P* = 0.02; *P*_BH_ = 0.19), and palmitate (*β* = −0.23; *P* = 0.03; *P*_BH_ = 0.19) were all negatively associated with motor scaled score, while petroselinic acid was negatively associated with language scaled score (*β* = −0.12; *P* = 0.047; *P*_BH_ = 0.69). All statistically significant results are also summarized via volcano plots in Fig. 4. Lastly, we conducted the same analysis using all 143 HILIC and 104 C18 metabolites, rather than the subset of ones significantly associated with feeding group and found the same trends in results. These analyses largely replicated the results in the main analyses and are summarized in Supplementary Figs. 5–8 as well as Supplementary File 2.

We also tested for associations between feeding groups and neurodevelopmental scores. Across both 1- and 6-month feeding groups, there were no statistically significant differences between the cognitive, motor, and language scaled scores of each feeding group. At 1-month, the exclusively breastmilk-fed group had the highest cognitive (Only breastmilk: *β* = 0.35; *P* = 0.62; Over 50% breastmilk: *β* = −0.56; *P* = 0.41), motor (Only breastmilk: *β* = 0.60; *P* = 0.53; Over 50% breastmilk: *β* = 0.41; *P* = 0.65), and language scaled scores (Only breastmilk: *β* = 0.08; *P* = 0.94; Over 50% breastmilk: *β* = −0.59; *P* = 0.54) compared to infants that received a majority of formula. At 6-months, the majority breastmilk group had higher Bayley’s scores across all three scoring domains, but these differences were not statistically significant, apart from motor scaled scores. Again, compared to infants receiving mostly formula, the majority breastmilk-fed group had a higher cognitive (*β* = 0.94; *P* = 0.11), motor (*β* = 1.63; *P* = 0.046), and language scaled scores (*β* = 0.59; *P* = 0.48).

## DISCUSSION

Using novel high-resolution metabolomics and well-characterized dietary and neurodevelopmental data collected across the first 2 years of life, this study explores the relationship between early life feeding patterns and the fecal metabolome in infants at 1- and 6-months old, and for the first time, ties these findings to neurodevelopmental outcomes at 2 years of age. Overall, we found that feeding groups were associated with 82 fecal metabolites and that 14 of these feeding-associated metabolites were also linked with neurodevelopmental outcomes at 2 years of age. To our knowledge, this is the first study to examine groups of infants by different amounts of mixed breastmilk and formula feedings and their associations with the infant fecal metabolome while including complementary solid foods at 6-months. Similarly, this is the first investigation to find evidence that feeding-specific metabolites are associated with neurodevelopment outcomes in early life. These findings suggest that early life feeding patterns have the potential to impact the infant fecal metabolome, which has implications for optimizing early life brain development.

Among the most pronounced and consistent findings was that infant feeding group in the first 6 months of life was associated both with the overall composition and with individual metabolites of the fecal metabolome. For example, we found that feeding group explained roughly 4–8% of the variability across samples. In comparison, our previous work found that 29–30% of the variation in the infant metabolome was explained by intra-individual variability in the first two years of life, while age accounted for 6–7% of variability during this time period^41^. This suggests that while intra-individual variability is the largest factor influencing the infant fecal metabolome, age and feeding patterns also substantially influence the fecal metabolome. Overall, our findings show that fecal metabolites at 1- and 6-months were associated with higher breastmilk and formula feedings per day, which is largely consistent with results from previous studies that examined either exclusive breastmilk or formula feeding. For example, we found that fecal cholesterol was positively associated with breastmilk feedings at 1- and 6-months. Breastmilk has higher cholesterol content than formula and may also induce synthesis of cholesterol through nutritional programming^49^. Conversely, formula has higher levels of plant-based oils^49,50^, which may explain why we observed higher levels of fecal palmitate and petroselinic/elaidic acid in infants fed formula for 50% or more of feedings. We also found that kynurenine intensity was higher in infants fed more breastmilk; similar findings from other infant fecal metabolome studies show that kynurenine abundance is higher in exclusively breastfed versus exclusively formula fed infants^46^. Likewise, kynurenine declines with infant age, which aligns with breastfeeding trajectories in early life^41^. We found that laurate levels were lowest in infants fed more breastmilk, which is in agreement with previous work that has noted that laurate is lower in breastmilk fed compared to formula fed infants^51^, with laurate even serving as a biomarker for formula feeding in another previous study^52^. Lastly, we found higher levels of the metabolite 4-pyridoxate with increasing formula feeding, which was also observed in exclusively formula fed compared to breastfed infants^42^. Collectively, these findings indicate that while exclusive breast feeding may not always be possible, increasing the proportions of breastmilk relative to formula may have beneficial impacts on the infant fecal metabolome.

While our primary aim was to determine if infant feeding patterns were associated with the infant fecal metabolome at 1-and 6-months of age, we additionally sought to determine if feeding-associated fecal metabolites were also associated with neurodevelopmental outcomes. Overall, we identified 14 feeding-associated metabolites that were linked with neurodevelopmental outcomes at 2 years of age. Specifically, except for caffeine, all breastmilk-associated metabolites were positively associated with language, motor, and cognitive scaled scores. Prenatal caffeine exposure has been previously reported to be associated with lower neurodevelopment scores at 6–7 years of age^53^. While typical consumption of caffeine (for example, up to 3 cups of coffee/day) is generally still considered safe for lactating mothers^54^, consumption above this level could cause caffeine to accumulate in an infant’s system, causing symptoms of caffeine stimulation^55^. Formula associated metabolites were negatively associated with neurodevelopmental scores at 2 years of age. Results from the current study suggest that these beneficial effects may be partly attributable to the specific metabolites, including lysoPC(16:0), cholesterol, and eicosadienoic acid. While fecal metabolites may be obtained directly from feeding and diet^56^, they may also be obtained through metabolic transformation by gut bacteria^57,58^. Supporting this hypothesis, the gut microbiome and bacterial-derived metabolites have been associated in murine models with brain function and development^59–61^.

Several studies have found that breastfeeding compared with formula feeding during infancy is associated with enhanced maturation of the central nervous system^29^ and earlier acquisition of key developmental milestones, including language and motor skills, and with overall higher cognitive development scoress^5,27,28^. We found that lysoPC(16:0) was positively associated with breastmilk feedings at 1-month and with language scaled scores at 2 years. LysoPC(16:0) is the preferred pathway of carrying DHA to the brain^62^, which is a long-chain polyunsaturated fatty acid that may have a positive impact on infant neurodevelopment^63,64^. We also found that cholesterol was positively associated with 1-month breastmilk consumption and higher language and with cognitive Bayley scaled scores at age 2 years. In early life, cholesterol is synthesized in the central nervous system during the first weeks following birth, and is implicated with many important neurodevelopmental processes, including synaptogenesis and myelination)^65^. Previous work has shown that children with higher dietary cholesterol intake perform better in cognitive developmental tests compared to those with the lowest consumption of dietary cholesterol^66^.

In contrast, formula feeding compared with breastfeeding during infancy has been previously implicated with poorer neurodevelopmental outcomes, including lower cognitive developmental scores^67^, and later completion of motor development milestones in mixed fed versus exclusively breastfed infants^68^. In this study, cadaverine was significantly associated with increased formula consumption at 1-month and lower language Bayley scaled scores at 2 years. Cadaverine is a common metabolite and contaminant present in infant formula^69^. It is classified as a biogenic amine, compounds known to elicit toxicological effects at high concentrations^69,70^. While no work has assessed the health effects of cadaverine in infants, increased levels of cadaverine have been observed in adults with ulcerative colitis^71^ and in children suffering from nutrient malabsorption conditions (e.g., cystic fibrosis, short bowel syndrome)^72^. While formula typically contains what is considered safe levels of biogenic amines^69^, future study in infants is warranted since they are more vulnerable to the health effects of contaminated foods. Other metabolites associated with formula included those derived from plant-based oils used to mimic fatty acid content present naturally in human milk. For instance, petroselinic acid/elaidic acid, which was associated with increased formula at 6-months and lower language scaled scores, is an industrially-derived trans fatty acid (TFA) produced by hydrogenation of vegetable oils^50^. Previous animal studies have demonstrated that brain DHA concentrations are reduced after high intakes of elaidic acid^73^, and that DHA deficiency during infancy delays brain development^74^. Additionally, a human study found increased TFAs were associated with unfavorable neurologic outcomes at 18-months^75^. We also found another plant-based oil associated metabolite, palmitate (a saturated fatty acid derived from palm oil), was associated with increased formula feedings at 6-months and lower motor scaled scores. In contrast with our findings, another study found formula supplemented with palmitate was associated with improved motor skills and higher levels of the beneficial bacteria *Bi*fi*dobacteria* in the infant gut due to a prebiotic effect^76^. These mixed findings suggest insufficient evidence regarding the role of palm oil/palmitate and whether it should be avoided in infant formula.

Findings from the current study suggest that formula-associated metabolites may have a greater impact on neurodevelopmental outcomes at 2 years of age compared with breastmilk-associated metabolites. At both 1- and 6-months, metabolites that were associated with increased formula feeding were also associated with lower neurodevelopment scores at 2 years. Conversely, metabolites associated with increased breast-feeding at only 1-month of age were associated with neurodevelopment outcomes. These differential effects of formula and breastfeeding may be attributable to the introduction of solid foods, which can disrupt the counter the protective effects of breastmilk^77^. For instance, which can disrupt the protective prior studies report that early solid food introduction is associated with increased allergenic responses in infants compared to exclusively breastmilk fed infants^77^. Additionally, the composition of the infant gut microbiome can change in both breastmilk and formula fed infants as solids foods are introduced^78^. For example, infants who were exclusively breastfed before solid food introduction have been shown to have a higher proportion of protective gut bacteria like *Bi*fi*dobacterium*, and a lower abundance of Bacteroidetes and Clostridiales – changes that are associated with altered immune functioning, increasing inflammation, and weight gain^79–81^. Therefore, increased breastmilk compared with formula feeding may provide the gut microbiome with a greater plasticity that eases the transition into solid foods^82^, which may partly explain why only formula-associated metabolites were negatively associated with neurodevelopmental outcomes following the introduction of solid foods. Overall, the timing of solid food introduction as well as the composition of those foods may have important implications for the protective effects of breastmilk feeding later in life. Moreover, all breastmilk is not created equal in the first 6 months of life; temporal variation in breastmilk composition may account for more associations at 1-month than 6-months of age. For example, the first form of breastmilk, colostrum, is higher in essential components like antibodies, protein, fatty acids, and other nutrients and growth factors than mature milk^83,84^. Although we do not have specific information regarding colostrum feeding, the strong associations seen at 1-month may reflect the positive neurodevelopmental effects of colostrum feeding shown in previous work^85^, which may persist but are no longer significant after 6-months. It is also pertinent to consider that formula feeding might disrupt the natural breast-feeding process by influencing milk supply. When infants are introduced to formula, it can lead to reduced demand for breastmilk, potentially impacting the stimulation necessary for sustained milk production. This alteration in breastfeeding patterns could further contribute to the variations observed in the composition of colostrum and mature milk, emphasizing the intricate interplay between feeding practices and the evolving nutritional content of breastmilk over the early stages of infant development. Understanding these dynamics is crucial for comprehensively interpreting the differences in infant gut metabolomics associated with various feeding patterns.

While this study had many strengths, including repeated sampling and comprehensive metabolomics profiling of the infant fecal metabolome from a well-established cohort of infants, it also has limitations worth noting. First, it focused on metabolites that were identified with Level 1 evidence, and therefore we characterized patterns and associations in a relatively limited number of metabolites, which may not capture the systemic alterations in the infant fecal metabolome across the first 6-months of life. Additionally, this study utilized an exclusively Latino cohort with exclusions for factors like preterm birth, low birth weight, cigarette smoking or recreational drug use, which may limit the generalizability of our findings. Stool samples were collected via OMNIGene GUT kits, which can limit the diversity of metabolites identified in comparison with other collections methods compared to immediate freezing^86^. However, other work shows that the biological effects, like individual variation, outweigh technical effects, such as collection method^87^. Further, many of the metabolites reported in this study have been previously observed in other infant metabolomic studies from varying populations^41,46,47,51,52^. Future studies should seek to incorporate the gut microbiome into fecal metabolomics to further explore microbial and metabolomic associations in the gut with dietary patterns, as gut bacteria likely play a key role in the composition of the fecal metabolome^58^. Finally, many factors may contribute to early life neurodevelopmental outcomes. While dietary patterns may play one role in shaping these outcomes, we cannot recognize the importance of other lifestyle factors (e.g., access to healthcare, social support, parenting practices, etc.) that also influence neurodevelopmental trajectories and outcomes in infants during early life.

Overall, this study showed that varying proportions of breastmilk or formula feedings are significantly associated with the composition of the infant fecal metabolome as well as individual metabolite intensities at both 1- and 6-months of age. Further, apart from caffeine, metabolites associated with more breastmilk feedings were associated with better neurodevelopmental performance at 2 years of age, while metabolites associated with more formula feedings were associated with worse neurodevelopmental scores. These findings suggest that increased breastfeeding, even in the context of Supplementary formula feeding, may have beneficial impacts on infant health and development.

## METHODS

### Study population

The Southern California Mother’s Milk Study is an ongoing, longitudinal cohort of 219 Latino mother-infant pairs who, beginning in 2016, were recruited from maternity clinics associated with the University of Southern California and Children’s Hospital Los Angeles, as described in detail in previous studies^41,88^. Individuals were eligible to participate in the Mother’s Milk Study if mothers were (1) ≥18 years old at time of delivery; (2) had a healthy, singleton birth; (3) enrolled in the study by 1-month postpartum; and (4) could read at a 5th-grade level in either Spanish or English. Potential participants were excluded if they had (1) any diagnoses known to impact mental/physical health, nutritional status, or metabolism; (2) were currently using tobacco or recreational drugs; (3) had infants who were self-reported by mothers to be preterm or low birth weight; or (4) had infants with clinically diagnosed fetal abnormalities. The Institutional Review Boards of the University of Southern California, Children’s Hospital Los Angeles, and the University of Colorado Boulder approved of the study procedures; all research was performed in accordance with the relevant guidelines and regulations. Written informed consent for the infants in this study was obtained from parents/legal guardians at time of enrollment.

### Study design

All participants were recruited from the Mother’s Milk Study between 2016–2017, a longitudinal cohort in which mothers and infants attended clinical visits at 1-, 6-, 12-, 18-, and 24-months postpartum. Initially, 219 mother-infant dyads enrolled in the Mother’s Milk cohort. A subset of 127 participants were selected based on completion of fecal sample collection at all timepoints to undergo fecal metabolomics analysis. Those individuals excluded from this analysis did not differ significantly from those who were included (Supplementary Table 1). SES was estimated using a modified version of the Hollingshead index^89^, as previously described, which ranged in possible value from 3–66^88,90^. Questionnaires were used to assess self-reported birth mode (vaginal or cesarean section), infant antibiotic exposure, and infant feeding practices.

### Infant feedings assessments

At 1- and 6-months, infant breastfeedings and formula feedings per day were based on questionnaire data with answer options of 0–1, 1, 2, 3, 4, 5, 6, 7, and ≥8 breast feedings per day. We assigned 0–1 as 0 feedings per day, 1–7 as their reported values, and ≥8 as eight feedings per day. Age of solid food introduction was assessed in months, e.g., a value of 5 indicates the infant began consuming solid foods when they were 5 months old. At 1- and 6-months, there were 113 infants with complete fecal metabolomic data at both timepoints, and of those, 112 infants had complete data on the number of feedings they received per day (one infant had no information recorded and was thus excluded from the analysis), see participant flow chart for more information (Supplementary Fig. 9). At 1-month, we defined feeding groups as (1) exclusively breastfed, meaning 100% of feedings per day were breastmilk and there was a 0 value for formula feedings per day, (*n* = 40), (2) breastfed >50% of feedings, (*n* = 46), or (3) formula fed ≥50% of feedings (*n* = 26). Importantly, as participants were recruited into the Mother’s Milk Study based on intention to breastfeed, there were very few participants who exclusively formula fed (*n* = 3), as such, we grouped based on levels of breastfeeding from most to least. These groupings were chosen based on total and within group sample size, as well as the distribution of breastmilk and formula feedings. We excluded infants at 6 months who either (a) were not eating solid foods yet (*n* = 16) or (b) reported solid food consumption, but had no data on supplementary milk or formula feedings (*n* = 10). This decreased the sample size at 6-months to 87 infants. At 6-months, feeding groups were defined as majority breastmilk (>50% of feedings, *n* = 41) or majority formula fed (≥50% of feedings, *n* = 46), both complemented by solid foods. The two sample groups at 1- and 6-months did not significantly differ in any characteristics except for age in days (Table 1).

### Neurodevelopmental assessments

Neurodevelopmental outcomes were assessed at 2 years of age using the Bayley Scales of Infant and Toddler Development-Third Edition (BSID-III)^91,92^. Trained research personnel administered the BSID-III under the supervision of an expert in child developmental assessment. Cognitive, motor, and language domains were assessed using BSID-III in an interactive examination lasting approximately 2 h. As there was a range in infant age at the 24-month visit (minimum age: 709 days; mean age: 735 days; maximum age: 799 days), scaled scores were used as the primary outcome, although composite scores (used to describe overall development in the relative domain) were also assessed. As a sensitivity analysis, we excluded infants who had difficulty completing the Bayley’s assessment (e.g., because of tiredness, crying, etc.; *n* = 8). However, because results were largely unchanged (Supplementary File 3), these infants were retained in the final analysis.

### Sample collection and extraction for high-resolution metabolomics

OMNIGene GUT kits were used to collect infant stool samples at 1-and 6-months of age. Untargeted high-resolution metabolomics analysis was carried out using established protocol by the Emory Clinical Biomarkers Laboratory, as previously described in detail^93,94^. Briefly, stool samples were first added to ice-cold acetonitrile to precipitate proteins, kept on ice for 30 min, centrifuged for 10 min at 14,000 g, and kept at 4 °C until analysis. Extractants were analyzed in triplicate using liquid chromatography coupled with high-resolution mass spectrometry (LC-HRMS) (Dionex Ultimate 3000, Thermo Scientific Orbitrap Fusion).

### Instrumentation and analytical conditions

Instrumentation methods for this analysis have been previously described in detail by Holzhausen et al^41^. In this study we used hydrophilic interaction liquid chromatography (HILIC) (Waters XBridge BEH Amide XP HILIC column; 2.1 × 50 mm^2^, 2.6 μm particle size) with positive electrospray ionization (ESI) and reverse phase (C18) chromatography (Higgins Targa C18 2.1 × 50 mm^2^, 3 μm particle size) with negative ESI. We conducted HILIC analyte separation using water, acetonitrile, and 2% formic acid mobile phases following the subsequent gradient elution. Our initial 1.5-min period consisted of 22.5% water, 75% acetonitrile, and 2.5% formic acid with a subsequent linear increase to 75% water, 22.5% acetonitrile, and 2.5% formic acid at 4 min, followed by a final hold for 1 min. We conducted analyte separation for the C18 chromatography column using water, acetonitrile, and 10 mM ammonium acetate mobile phases under the following gradient elution. The initial 1-min period consisted of 60% water, 35% acetonitrile, and 5% ammonium acetate with a subsequent linear increase to 0% water, 95% acetonitrile, and 5% ammonium acetate at 3 min with a final hold for the last 2 min. Mobile phase flow rate was 0.35 mL/min for the first minute and was increased to 0.4 mL/min for the last 4 min for the HILIC and C18 chromatography columns. LC-HRMS was run in full scan mode, with 120k resolution; the range of mass-to-charge ratio (m/z) was from 85 to 1275. Tuning parameters for sheath gas were 45 (arbitrary units) for positive ESI and 30 for negative ESI. For positive ESI, auxiliary gas was set to 25 (arbitrary units) and spray voltage was set at 3.5 kV. For negative ESI, auxiliary gas was set to 5, and spray voltage was set to −3.0 kV. Internal standards included pooled stool and standard reference materials for human metabolites in stool. We added these internal standards at the beginning and end of each 20-sample batch for quality control and standardization.

### Metabolite confidence and identification

We analyzed data from HILIC positive ESI and C18 negative ESI separately; raw files were converted to the .mzXML format. Two internal standards, which include pooled stool and standard reference material for human stool metabolites (NIST SRM 1950), were added at the beginning and the end of each batch of 20 samples for normalization, to control for background noise, batch evaluation, and post hoc quantification. Metabolomic signals (i.e., metabolic features) were then extracted and aligned using apLCMS^95^ with modification of xMSanalyzer^96^ for quality control and reduction of batch effects following instrument analysis^96,97^. Coefficients of variation (CV) of metabolic features were assessed as part of our quality control. Metabolic features whose intensity had CV > 30% were removed, then intensities of metabolic features were averaged across triplicates. Metabolic features which were detected in <10% of samples were excluded. Outliers were assessed visually using principal component analysis (PCA) of the log_2_ transformed feature intensities. In a sensitivity analysis, samples whose PCA score was >3 standard deviations for PC 1 or PC 2 were removed (not shown). There were no important differences in results, and as such these observations were not removed. Metabolic features were then annotated and confirmed using the Metabolomics Standards initiative criteria^98^. Level 1 confidence was assigned to metabolic features whose *m/z*, retention time, and extracted ion chromatograph matched the authentic standards analyzed with tandem mass spectrometry under identical conditions (within 10 ppm and 50 s; HILIC maximum retention time difference: 41.7, HILIC minimum difference: −48.1; C18 maximum retention time difference: 38.1, HILIC minimum difference: −49.1). In all analyses described below, we focus on these confirmed metabolites with Level 1 evidence.

### Statistical analysis

Descriptive statistics for key variables were performed on the full analytic data set of 112 participants at 1-month or 87 participants at 6-months of age. We used the {ggVenn} package in R to visualize how many metabolites were present in 50% of samples in each feeding grouping at each timepoint^99^. We performed PCA on log_2_ transformed feature intensities to visualize overall metabolomics profiles between feeding groupings within each timepoint of either 1- or 6-months; any metabolic features which were below the minimum level of detection, and were therefore missing were set to 0 before performing the PCA. We used permutational multivariate ANOVA (PERMANOVA) tests to explore how overall fecal metabolite intensities changed in relation to feeding groupings, using the “adonis2” function implemented by the {vegan} package in R and using Euclidian distance (permutations = 1000) while removing any missing values before performing the distance calculation^100^.

**Fig. 5:**
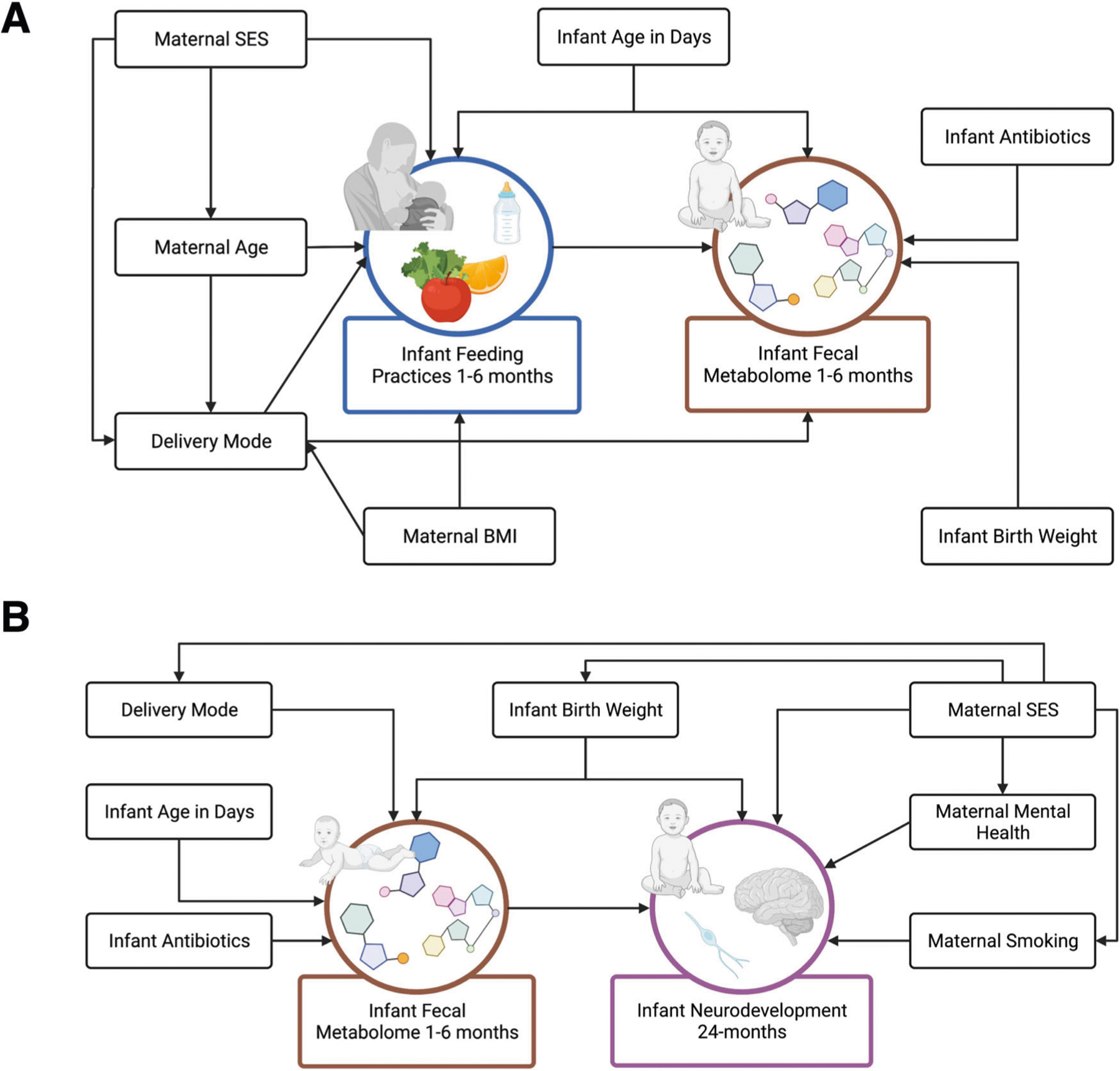
Directed acyclic graphs between infant feeding (exposure) and fecal metabolome (outcome), and fecal metabolome (exposure) and infant neurodevelopment (outcome). Directed acyclic graphs (DAGs) were developed based on causal relationships determined from review of relevant literature using (**A**) infant feeding practices at 1- and 6-months as the exposure, and the infant fecal metabolome at 1- and 6-months as the outcome and (**B**) the infant fecal metabolome at 1- and 6-months as the exposure and infant neurodevelopment at 24-months as the outcome. Figures were created with BioRender.com.

Linear models were used to estimate the associations of the log_2_ transformed intensity of each confirmed Level 1 metabolite with infant feeding groupings at 1- and 6-month timepoints. Models included adjustments for infant age in days and mode of delivery based on a Directed Acyclic Graph (DAG) (Fig. 5A), with results adjusted for multiple testing using the Benjamini-Hochberg procedure at *P*_BH_ < 0.05^101^. Boxplots were used to visualize the intensity of selected confirmed metabolites associated with infant age in days in the HILIC and C18 chromatography columns by feeding groupings.

We ran metabolome-wide association studies using linear models to estimate the relationship between the log_2_ transformed intensity of each confirmed metabolite that was also significantly associated with feeding and neurodevelopmental outcomes (Bayley scores) at 24-months. We also ran the same models to estimate relationships between all confirmed metabolites as an untargeted approach. Models included adjustments for infant birthweight and mode of delivery based on a DAG (Fig. 5B), with results adjusted for multiple testing using the Benjamini-Hochberg procedure at *P*_BH_ ≤ 0.20. Considering the exploratory nature of this study, we chose a significance level of *P*_BH_ < 0.20. By selecting a 20% false discovery rate, we aimed to capture biologically meaningful associations between feeding-associated gut metabolites and neurodevelopmental outcomes. Volcano plots were used to visualize which metabolites were significantly associated with each Bayley score measure and were also significantly associated with feeding groupings from the previous linear models. We also ran linear models to estimate the relationship between neurodevelopmental outcomes and infant feeding groupings at 1- and 6-months postpartum. These models included adjustment for maternal SES based on a DAG.

## Supplementary Material

Checklist

SFile 1

SFile 2

SFile 3

STable and Fig

## Figures and Tables

**Table 1.** Characteristics of mother-infant dyads from the Southern California Mother’s Milk Study, 2016–2019.

	1-Month Mean ± SD or *N*, % *N* = 112	6-Months Mean ± SD or *N*, % *N* = 87	*P*-value
*Maternal Characteristics*			
Age at visit (years)	29.0 ± 6.3	29.4 ±6.3	0.65
Socioeconomic status (SES)	26.5 ± 12.0	26.2 ± 12.4	0.89
Pre-pregnancy BMI (kg/m^2^)	28.5 ± 5.8	28.9 ± 6.1	0.61
*Infant Characteristics*			
Age (days)	32.5 ± 3.4	185.9 ± 8.1	<0.001
Sex (female, male, %female)	60, 52, 53.6%	48, 39, 55.2%	0.94
Mode of Delivery (c-section, vaginal, %c-section)	31, 81, 27.7%	23, 64, 26.4%	0.97
Age of solid foods (months)	-	5.4 ± 0.7	-
Antibiotics (yes, no, %yes)	12, 100, 10.7%	10, 77, 11.6%	1.0
Birth weight (kg)	3.4 ± 0.4	3.4 ± 0.4	0.58
Birth length (cm)	50.4 ± 2.4	50.2 ± 2.6	0.94

Characteristics of 112 and 87 Latino mother-infant dyads at 1-month and 6-months, respectively. Data reported are mean and standard deviation (SD) unless otherwise noted. For continuous variables, independent *t* tests were used to test for differences between those who were included in the 1-month analysis and those in the 6-month analyses, unless *p* value is denoted with an asterisk (*), in which case a Wilcoxon rank-sum test was used. For categorical variables, Chi-square tests were used to test for differences between those included at 1- or 6-months.

*SES* socioeconomic status, based on Hollingshead Index (range: 3–66, lower scores indicate a lower social status), *BMI* body mass index.

**Table 2.** Top ten confirmed metabolites detected by HILIC and C18 chromatography columns that were associated with infant feeding at 1-month of age.

HILIC Column (1-Month)			C18 Column (1-Month)		
Metabolite	Positive Associations with Feeding Type	*P*_BH_	Metabolite	Positive Associations with Feeding Type	*P*_BH_
KYNURENINE	Breastmilk	8.3 × 10^−7^	HEPTADECANOATE	Breastmilk	2.3 × 10^−9^
CHOLESTEROL	Breastmilk	4.6 × 10^−6^	EICOSADIENOIC ACID	Breastmilk	6.7 × 10^−5^
LYSOPC(16:0)	Breastmilk	1.2 × 10^−3^	LAURATE	Formula	2.3 × 10^−10^
4-PYRIDOXATE	Formula	4.5 × 10^−8^	HYPOXANTHINE	Formula	4.2 × 10^−8^
GLYCERIC ACID	Formula	7.5 × 10^−8^	GLYCERATE	Formula	1.0 × 10^−6^
DOPA	Formula	3.7 × 10^−5^	OMEGA-HYDROXYDODECANOIC ACID	Formula	1.0 × 10^−6^
GLUCONO-1,5-LACTONE	Formula	8.3 × 10^−5^	MYRISTATE	Formula	4.6 × 10^−6^
GLUTAMIC ACID/N-METHYL-ASPARTATE	Formula	9.3 × 10^−5^	LYSINE	Formula	1.5 × 10^−5^
THYMIDINE	Formula	2.4 × 10^−3^	PALMITATE	Formula	2.2 × 10^−4^
PROLINE	Formula	2.6 × 10^−3^	RAC-GLYCEROL 1-MYRISTATE	Formula	3.0 × 10^−4^

Top ten confirmed metabolites detected by HILIC and C18 chromatography columns that were most significantly associated with infant feeding at 1-month of age, which was based on the results from linear models that adjusted for infant age in days and mode of delivery. Results from these models were adjusted using the Benjamini-Hochberg (BH) procedure. All metabolites identified were positively associated with feeding type as indicated (i.e., breastmilk or formula). There were 35 metabolites significantly associated with infant feeding after adjustment for multiple testing in the HILIC chromatography column and 22 in the C18 chromatography column at 1-month.

**Table 3.** Top ten confirmed metabolites detected by HILIC and C18 chromatography columns that were associated with infant feeding at 6-months of age.

HILIC Column (6-Months)			C18 Column (6-Months)		
Metabolite	Positive Associations with Feeding Type	*P*_BH_	Metabolite	Positive Associations with Feeding Type	*P*_BH_
CHOLESTEROL	Breastmilk	2.9 × 10^−6^	ALPHA-AMINOADIPATE/N-METHYL-GLUTAMATE	Breastmilk	1.6 × 10^−4^
3-METHYLADENINE	Breastmilk	9.3 × 10^−3^	METHYL VANILLATE/HOMOVANILLATE	Breastmilk	1.6 × 10^−3^
KYNURENINE	Breastmilk	9.3 × 10^−3^	6-DEOXY-GALACTOSE (FUCOSE)/RHAMNOSE	Breastmilk	3.7 × 10^−3^
GLUCOSAMINE/MANNOSAMINE	Breastmilk	2.3 × 10^−2^	LAURATE	Formula	1.4 × 10^−5^
N-ALPHA-ACETYL-ASPARAGINE	Breastmilk	4.3 × 10^−2^	PALMITATE	Formula	3.4 × 10^−5^
CYTOSINE	Breastmilk	6.4 × 10^−2^	MYRISTATE	Formula	8.8 × 10^−5^
4-PYRIDOXATE	Formula	1.3 × 10^−3^	RAC-GLYCEROL 1-MYRISTATE	Formula	8.8 × 10^−5^
GLYCERIC ACID	Formula	1.3 × 10^−2^	PETROSELINIC ACID/ELAIDIC ACID	Formula	1.8 × 10^−3^
METHYLHIPPURATE	Formula	2.3 × 10^−2^	HYPOXANTHINE	Formula	9.3 × 10^−3^
GUANOSINE 5’-MONOPHOSPHATE	Formula	3.4 × 10^−2^	LINOLEATE	Formula	9.9 × 10^−3^

Top ten confirmed metabolites detected by HILIC and C18 chromatography columns that were most significantly associated with infant feeding at 1-month of age, which was based on the results from linear models that adjusted for infant age in days and mode of delivery. Results from these models were adjusted using the Benjamini-Hochberg (BH) procedure. All metabolites identified were positively associated with feeding type as indicated (i.e., breastmilk or formula). There are nine metabolites significantly associated with infant feeding after adjustment for multiple testing in the HILIC chromatography column and 16 in the C18 chromatography column at 6-months.

## Data Availability

The data are not publicly available since deposition of de-identified sequences into a public database was not included in the original informed consent for the Mother’s Milk Study. The data and analytic code that support the findings of this study are available upon reasonable request from the corresponding author, T.L.A.

## References

[1] Hanson LÅ, Korotkova M & Telemo E Breast-feeding, infant formulas, and the immune system. Ann. Allergy Asthma Immunol 90, 59–63 (2003). 12839115 10.1016/s1081-1206(10)61662-6

[2] Camacho-Morales A Breastfeeding contributes to physiological immune programming in the newborn. Front. Pediatr 9, 744104 (2021). 34746058 10.3389/fped.2021.744104 PMC8567139

[3] Forbes JD Association of exposure to formula in the hospital and subsequent infant feeding practices with gut microbiota and risk of overweight in the first year of life. JAMA Pediatr. 172, e181161 (2018). 29868719 10.1001/jamapediatrics.2018.1161 PMC6137517

[4] Hildebrand JS Breastfeeding associations with childhood obesity and body composition: findings from a racially diverse maternal–child Cohort. Child. Obes 18, 178–187 (2022). 34669515 10.1089/chi.2021.0138 PMC8982114

[5] Bar S, Milanaik R & Adesman A Long-term neurodevelopmental benefits of breastfeeding. Curr. Opin. Pediatr 28, 559 (2016). 27386975 10.1097/MOP.0000000000000389

[6] World Health Organization. Breastfeeding. https://www.who.int/health-topics/breastfeeding.

[7] CDC. Breastfeeding Report Card United States, 2022. https://www.cdc.gov/breastfeeding/data/reportcard.htm.

[8] Nguyen P Prelacteal and early formula feeding increase risk of infant hospitalization: a prospective cohort study. Arch. Dis. Child 105, 122–126 (2020). 31523040 10.1136/archdischild-2019-316937

[9] Koletzko B Infant Feeding and Later Obesity Risk. in Early Nutrition Programming and Health Outcomes in Later Life (eds. Koletzko B, Decsi T, Molnár D & de la Hunty A) 15–29 (Springer Netherlands, 2009). 10.1007/978-1-4020-9173-5_2.

[10] Decsi T, Thiel I & Koletzko B Essential fatty acids in full term infants fed breast milk or formula. Arch. Dis. Child. Fetal Neonatal Ed 72, F23–F28 (1995). 7743279 10.1136/fn.72.1.f23 PMC2528425

[11] Cunnane SC, Francescutti V, Brenna JT & Crawford MA Breast-fed infants achieve a higher rate of brain and whole body docosahexaenoate accumulation than formula-fed infants not consuming dietary docosahexaenoate. Lipids 35, 105–111 (2000). 10695931 10.1007/s11745-000-0501-6

[12] van Dongen KCW, Ioannou A, Wesseling S, Beekmann K & Belzer C Differences in gut microbial fructoselysine degradation activity between breastfed and formula-fed infants. FEMS Microbiol. Ecol 99, fiac145 (2023). 10.1093/femsec/fiac145 PMC9749803 36442156

[13] Awan I A pilot study exploring temporal development of gut microbiome/metabolome in breastfed neonates during the first week of life. Pediatr. Gastroenterol. Hepatol. Nutr 26, 99–115 (2023). 36950061 10.5223/pghn.2023.26.2.99 PMC10025571

[14] Oddy WH Early infant feeding and adiposity risk: from infancy to adulthood. Ann. Nutr. Metab 64, 262–270 (2014). 25300269 10.1159/000365031

[15] Koleva PT, Bridgman SL & Kozyrskyj AL The infant gut microbiome: evidence for obesity risk and dietary intervention. Nutrients 7, 2237–2260 (2015). 25835047 10.3390/nu7042237 PMC4425142

[16] Nguyen QP Associations between the gut microbiome and metabolome in early life. BMC Microbiol. 21, 238 (2021). 34454437 10.1186/s12866-021-02282-3 PMC8400760

[17] De Filippo C Impact of diet in shaping gut microbiota revealed by a comparative study in children from Europe and rural Africa. Proc. Natl. Acad. Sci. USA 107, 14691–14696 (2010). 20679230 10.1073/pnas.1005963107 PMC2930426

[18] Voreades N, Kozil A & Weir TL Diet and the development of the human intestinal microbiome. Front. Microbiol 5, 494 (2014). 25295033 10.3389/fmicb.2014.00494 PMC4170138

[19] Conta G Longitudinal multi-omics study of a mother-infant dyad from breastfeeding to weaning: an individualized approach to understand the interactions among diet, fecal metabolome, and microbiota composition. Front. Mol. Biosci 8, 688440 (2021). 34671642 10.3389/fmolb.2021.688440 PMC8520934

[20] Zierer J The fecal metabolome as a functional readout of the gut microbiome. Nat. Genet 50, 790–795 (2018). 29808030 10.1038/s41588-018-0135-7 PMC6104805

[21] Conlon MA & Bird AR The Impact of Diet and Lifestyle on Gut Microbiota and Human Health. Nutrients 7, 17–44 (2015). 10.3390/nu7010017 PMC4303825 25545101

[22] Kelly D, King T & Aminov R Importance of microbial colonization of the gut in early life to the development of immunity. Mutat. Res 622, 58–69 (2007). 17612575 10.1016/j.mrfmmm.2007.03.011

[23] Harmsen HJM Analysis of intestinal flora development in breast-fed and formula-fed infants by using molecular identification and detection methods. J. Pediatr. Gastroenterol. Nutr 30, 61 (2000). 10630441 10.1097/00005176-200001000-00019

[24] Caudill MA Pre- and postnatal health: evidence of increased choline needs. J. Am. Diet. Assoc 110, 1198–1206 (2010). 20656095 10.1016/j.jada.2010.05.009

[25] Zeisel SH & Blusztajn JK Choline and human nutrition. Ann. Rev. Nutr 14, 269–296 (1994). 7946521 10.1146/annurev.nu.14.070194.001413

[26] Zeisel SH Choline: critical role during fetal development and dietary requirements in adults. Annu. Rev. Nutr 26, 229–250 (2006). 16848706 10.1146/annurev.nutr.26.061505.111156 PMC2441939

[27] Dee DL, Li R, Lee L-C & Grummer-Strawn LM Associations between breastfeeding practices and young children’s language and motor skill development. Pediatrics 119, S92–S98 (2007). 17272591 10.1542/peds.2006-2089N

[28] Julvez J A cohort study on full breastfeeding and child neuropsychological development: the role of maternal social, psychological, and nutritional factors. Dev. Med. Child. Neurol 56, 148–156 (2014). 24116864 10.1111/dmcn.12282

[29] Khedr E, Farghaly W, Amry SE-D & Osman A Neural maturation of breastfed and formula-fed infants. Acta Paediatr. 93, 734–738 (2004). 15244219 10.1111/j.1651-2227.2004.tb03011.x

[30] Cusick SE & Georgieff MK The role of nutrition in brain development: the golden opportunity of the “First 1000 Days”. J. Pediatr 175, 16–21 (2016). 27266965 10.1016/j.jpeds.2016.05.013 PMC4981537

[31] Sordillo JE Association of the infant gut microbiome with early childhood neurodevelopmental outcomes: an ancillary study to the VDAART randomized clinical trial. JAMA Netw. Open 2, e190905 (2019). 30901046 10.1001/jamanetworkopen.2019.0905 PMC6583279

[32] Tamana SK Bacteroides-dominant gut microbiome of late infancy is associated with enhanced neurodevelopment. Gut Microbes 13, 1930875 (2021). 10.1080/19490976.2021.1930875 PMC8210878 34132157

[33] Rothenberg SE Neurodevelopment correlates with gut microbiota in a cross-sectional analysis of children at 3 years of age in rural China. Sci. Rep 11, 7384 (2021). 33795717 10.1038/s41598-021-86761-7 PMC8016964

[34] Laghi L Are fecal metabolome and microbiota profiles correlated with autism severity? A cross-sectional study on ASD preschoolers. Metabolites 11, 654 (2021). 34677369 10.3390/metabo11100654 PMC8539853

[35] Needham BD Plasma and fecal metabolite profiles in autism spectrum disorder. Biol. Psychiatry 89, 451–462 (2021). 33342544 10.1016/j.biopsych.2020.09.025 PMC7867605

[36] Horta BL, Loret de Mola C & Victora CG Breastfeeding and intelligence: a systematic review and meta-analysis. Acta Paediatr. 104, 14–19 (2015). 26211556 10.1111/apa.13139

[37] Kramer MS Breastfeeding and child cognitive development: new evidence from a large randomized trial. Arch. Gen. Psychiatry 65, 578–584 (2008). 18458209 10.1001/archpsyc.65.5.578

[38] Knickmeyer RC A structural mri study of human brain development from birth to 2 years. J. Neurosci 28, 12176–12182 (2008). 19020011 10.1523/JNEUROSCI.3479-08.2008 PMC2884385

[39] Tau GZ & Peterson BS Normal development of brain circuits. Neuropsychopharmacol. 35, 147–168 (2010). 10.1038/npp.2009.115 PMC3055433 19794405

[40] Diaz Heijtz R Fetal, neonatal, and infant microbiome: perturbations and subsequent effects on brain development and behavior. Sem. Fetal Neonatal Med 21, 410–417 (2016). 10.1016/j.siny.2016.04.012 27255860

[41] Holzhausen EA Longitudinal profiles of the fecal metabolome during the first 2 years of life. Sci. Rep 13, 1886 (2023). 36732537 10.1038/s41598-023-28862-z PMC9895434

[42] Li N Distinct gut microbiota and metabolite profiles induced by delivery mode in healthy Chinese infants. J. Proteom 232, 104071 (2021). 10.1016/j.jprot.2020.104071 33307251

[43] Patton L Antibiotics effects on the fecal metabolome in preterm infants. Metabolites 10, 331 (2020). 32823682 10.3390/metabo10080331 PMC7464203

[44] Lu S, Huang Q, Wei B & Chen Y Effects of β-lactam antibiotics on gut microbiota colonization and metabolites in late preterm infants. Curr. Microbiol 77, 3888–3896 (2020). 32970172 10.1007/s00284-020-02198-7

[45] Parolin C, Zhu W & Zhu J Editorial: metabolomics of human microbiome studies: recent advances in methods and applications. Front. Mol. Biosci 8, 800337 (2021). 34917651 10.3389/fmolb.2021.800337 PMC8670226

[46] Brink LR Neonatal diet alters fecal microbiota and metabolome profiles at different ages in infants fed breast milk or formula. The American Journal of Clinical Nutrition 111, 1190–1202 (2020). 32330237 10.1093/ajcn/nqaa076 PMC7266684

[47] Sillner N Longitudinal profiles of dietary and microbial metabolites in formula- and breastfed infants. Front. Mol. Biosci 8, 660456 (2021). 34124150 10.3389/fmolb.2021.660456 PMC8195334

[48] He X Metabolic phenotype of breast-fed infants, and infants fed standard formula or bovine MFGM supplemented formula: a randomized controlled trial. Sci. Rep 9, 339 (2019). 30674917 10.1038/s41598-018-36292-5 PMC6344597

[49] Zubin Maslov P, Hill JA, Lüscher TF & Narula J High-sugar feeding and increasing cholesterol levels in infants. Eur. Heart J 42, 1132–1135 (2021). 33326580 10.1093/eurheartj/ehaa868

[50] Chisaguano AM Elaidic, vaccenic, and rumenic acid status during pregnancy: association with maternal plasmatic LC-PUFAs and atopic manifestations in infants. Pediatr. Res 76, 470–476 (2014). 25119335 10.1038/pr.2014.119

[51] Rodriguez-Herrera A Early-life fecal microbiome and metabolome dynamics in response to an intervention with infant formula containing specific prebiotics and postbiotics. Am. J. Physiol. Gastrointest. Liver Physiol 322, G571–G582 (2022). 35348015 10.1152/ajpgi.00079.2021 PMC9109790

[52] Wang M, Li M, Chapkin RS, Ivanov I & Donovan SM Fecal microbiome and metabolites differ between breast and formula-fed human infants. FASEB J. 27, 850.4–850.4 (2013).

[53] Laue HE In Utero Exposure to Caffeine and Acetaminophen, the Gut Microbiome, and Neurodevelopmental Outcomes: A Prospective Birth Cohort Study. Int. J. Environ. Res. Public Health 19, 9357 (2022). 35954712 10.3390/ijerph19159357 PMC9367926

[54] Jeong G, Park SW, Lee YK, Ko SY & Shin SM Maternal food restrictions during breastfeeding. Korean J. Pediatr 60, 70–76 (2017). 28392822 10.3345/kjp.2017.60.3.70 PMC5383635

[55] Mohrbacher N & Stock J The breastfeeding answer book. Rev La Leche League International. (1997).

[56] Russell WR High-protein, reduced-carbohydrate weight-loss diets promote metabolite profiles likely to be detrimental to colonic health. Am. J. Clin. Nutr 93, 1062–1072 (2011). 21389180 10.3945/ajcn.110.002188

[57] The Human Microbiome Project Consortium Structure, function and diversity of the healthy human microbiome. Nature 486, 207–214 (2012). 22699609 10.1038/nature11234 PMC3564958

[58] Karu N A review on human fecal metabolomics: methods, applications, and the human fecal metabolome database. Anal. Chim. Acta 1030, 1–24 (2018). 30032758 10.1016/j.aca.2018.05.031

[59] Braniste V The gut microbiota influences blood-brain barrier permeability in mice. Sci. Transl. Med 6, 263ra158–263ra158 (2014). 10.1126/scitranslmed.3009759 PMC4396848 25411471

[60] Chu C The microbiota regulate neuronal function and fear extinction learning. Nature 574, 543–548 (2019). 31645720 10.1038/s41586-019-1644-y PMC6818753

[61] Erny D Host microbiota constantly control maturation and function of microglia in the CNS. Nat. Neurosci 18, 965–977 (2015). 26030851 10.1038/nn.4030 PMC5528863

[62] Lo Van A Mechanisms of DHA transport to the brain and potential therapy to neurodegenerative diseases. Biochimie 130, 163–167 (2016). 27496085 10.1016/j.biochi.2016.07.011

[63] Basak S, Mallick R & Duttaroy AK Maternal docosahexaenoic acid status during pregnancy and its impact on infant neurodevelopment. Nutrients 12, 3615 (2020). 33255561 10.3390/nu12123615 PMC7759779

[64] Bazan NG Omega-3 fatty acids, pro-inflammatory signaling and neuroprotection. Curr. Opin. Clin. Nutr. Metab. Care 10, 136 (2007). 17285000 10.1097/MCO.0b013e32802b7030

[65] Hussain G Role of cholesterol and sphingolipids in brain development and neurological diseases. Lipids Health Dis. 18, 26 (2019). 30683111 10.1186/s12944-019-0965-z PMC6347843

[66] Wright RO Apolipoprotein E Genotype Predicts 24-Month Bayley Scales Infant Development Score. Pediatr. Res 54, 819–825 (2003). 12930912 10.1203/01.PDR.0000090927.53818.DE

[67] Anderson JW, Johnstone BM & Remley DT Breast-feeding and cognitive development: a meta-analysis. Am. J. Clin. Nutr 70, 525–535 (1999). 10500022 10.1093/ajcn/70.4.525

[68] Dewey KG, Cohen RJ, Brown KH & Rivera LL Effects of exclusive breastfeeding for four versus six months on maternal nutritional status and infant motor development: results of two randomized trials in Honduras. J. Nutr 131, 262–267 (2001). 11160544 10.1093/jn/131.2.262

[69] Spizzirri UG, Puoci F, Iemma F & Restuccia D Biogenic amines profile and concentration in commercial milks for infants and young children. Food Addit. Contam.: Part A 36, 337–349 (2019). 10.1080/19440049.2018.1563306 30722764

[70] Hazards (BIOHAZ), E. P. on B. Scientific Opinion on risk based control of biogenic amine formation in fermented foods. EFSA J. 9, 2393 (2011).

[71] Le Gall G Metabolomics of fecal extracts detects altered metabolic activity of gut microbiota in ulcerative colitis and irritable bowel syndrome. J. Proteome Res 10, 4208–4218 (2011). 21761941 10.1021/pr2003598

[72] Forget P, Sinaasappel M, Bouquet J, Deutz NEP & Smeets C Fecal polyamine concentration in children with and without nutrient malabsorption. J. Pediatr. Gastroenterol. Nutr 24, 285 (1997). 9138174 10.1097/00005176-199703000-00010

[73] Wauben IPM, Xing H-C, McCutcheon D & Wainwright PE Dietary trans fatty acids combined with a marginal essential fatty acid status during the pre- and postnatal periods do not affect growth or brain fatty acids but may alter behavioral development in B6D2F2 mice. J. Nutr 131, 1568–1573 (2001). 11340117 10.1093/jn/131.5.1568

[74] Yehuda S, Rabinovitz S & Mostofsky DI Essential fatty acids and the brain: From infancy to aging. Neurobiol. Aging 26, 98–102 (2005). 16226347 10.1016/j.neurobiolaging.2005.09.013

[75] Bouwstra H Neurologic condition of healthy term infants at 18 months: positive association with venous umbilical DHA Status and negative association with umbilical trans-fatty acids. Pediatr Res 60, 334–339 (2006). 16857765 10.1203/01.pdr.0000233043.16674.1d

[76] Wu W Neurodevelopmental Outcomes and gut bifidobacteria in term infants fed an infant formula containing high sn-2 palmitate: a cluster randomized clinical trial. Nutrients 13, 693 (2021). 33671493 10.3390/nu13020693 PMC7926808

[77] Tarini BA, Carroll AE, Sox CM & Christakis DA Systematic review of the relationship between early introduction of solid foods to infants and the development of allergic disease. Arch. Pediatr. Adolesc. Med 160, 502–507 (2006). 16651493 10.1001/archpedi.160.5.502

[78] Palmer C, Bik EM, DiGiulio DB, Relman DA & Brown PO Development of the human infant intestinal microbiota. PLoS Biol. 5, e177 (2007). 17594176 10.1371/journal.pbio.0050177 PMC1896187

[79] Thompson AL, Monteagudo-Mera A, Cadenas MB, Lampl ML & Azcarate-Peril MA Milk- and solid-feeding practices and daycare attendance are associated with differences in bacterial diversity, predominant communities, and metabolic and immune function of the infant gut microbiome. Front. Cell. Infect 5, 3 (2015). 10.3389/fcimb.2015.00003 PMC4318912 25705611

[80] Nauta AJ, Ben Amor K, Knol J, Garssen J & van der Beek E Relevance of pre- and postnatal nutrition to development and interplay between the microbiota and metabolic and immune systems. Am. J. Clin. Nutr 98, 586S–593S (2013). 23824726 10.3945/ajcn.112.039644

[81] Greiner T & Bäckhed F Effects of the gut microbiota on obesity and glucose homeostasis. Trends Endocrinol. Metab 22, 117–123 (2011). 21353592 10.1016/j.tem.2011.01.002

[82] Matamoros S, Gras-Leguen C, Le Vacon F, Potel G & de La Cochetiere M-F Development of intestinal microbiota in infants and its impact on health. Trends Microbiol. 21, 167–173 (2013). 23332725 10.1016/j.tim.2012.12.001

[83] Uruakpa FO, Ismond MAH & Akobundu ENT Colostrum and its benefits: a review. Nutr. Res 22, 755–767 (2002).

[84] Kobata R High levels of growth factors in human breast milk. Early Hum. Dev 84, 67–69 (2008). 17716837 10.1016/j.earlhumdev.2007.07.005

[85] Guxens M Breastfeeding, long-chain polyunsaturated fatty acids in colostrum, and infant mental development. Pediatrics 128, e880–e889 (2011). 21930546 10.1542/peds.2010-1633 PMC9923846

[86] Wang Z Comparison of fecal collection methods for microbiome and metabolomics studies. Front. Cell. Infect. Microbiol 8, 301 (2018). 30234027 10.3389/fcimb.2018.00301 PMC6127643

[87] Guan H Comparison of fecal collection methods on variation in gut metagenomics and untargeted metabolomics. mSphere 6, e00636–21 (2021). 34523982 10.1128/mSphere.00636-21 PMC8550109

[88] Patterson WB Prenatal exposure to ambient air pollutants and early infant growth and adiposity in the Southern California Mother’s Milk Study. Environ. Health 20, 67 (2021). 34090448 10.1186/s12940-021-00753-8 PMC8180163

[89] Hollingshead AB Four factor index of social status. Yale Journal of Sociology. (1975).

[90] Morgan ZEM Prenatal exposure to ambient air pollution is associated with neurodevelopmental outcomes at 2 years of age. Environ. Health 22, 11 (2023). 36694159 10.1186/s12940-022-00951-y PMC9872424

[91] Albers CA & Grieve AJ Review of Bayley scales of infant and toddler development-third edition. J. Psychoeduc. Assess 25, 180–190 (2007).

[92] Rosario CD, Slevin M, Molloy EJ, Quigley J & Nixon E How to use the Bayley scales of infant and toddler development. Arch. Dis. Child. Educ. Practice 106, 108–112 (2021). 10.1136/archdischild-2020-319063 32859738

[93] Go Y-M Reference standardization for mass spectrometry and high-resolution metabolomics applications to exposome research. Toxicol. Sci 148, 531–543 (2015). 26358001 10.1093/toxsci/kfv198 PMC4675836

[94] Liang D Use of high-resolution metabolomics for the identification of metabolic signals associated with traffic-related air pollution. Environ. Int 120, 145–154 (2018). 30092452 10.1016/j.envint.2018.07.044 PMC6414207

[95] Yu T, Park Y, Johnson JM & Jones DP apLCMS—adaptive processing of high-resolution LC/MS data. Bioinformatics 25, 1930–1936 (2009). 19414529 10.1093/bioinformatics/btp291 PMC2712336

[96] Uppal K xMSanalyzer: automated pipeline for improved feature detection and downstream analysis of large-scale, non-targeted metabolomics data. BMC Bioinform. 14, 15 (2013). 10.1186/1471-2105-14-15 PMC3562220 23323971

[97] Yu T & Jones DP Improving peak detection in high-resolution LC/MS metabolomics data using preexisting knowledge and machine learning approach. Bioinformatics 30, 2941–2948 (2014). 25005748 10.1093/bioinformatics/btu430 PMC4184266

[98] Sumner LW Proposed minimum reporting standards for chemical analysis. Metabolomics 3, 211–221 (2007). 24039616 10.1007/s11306-007-0082-2 PMC3772505

[99] Yan L & Yan ML Package “ggvenn”. Available online: https://cren.r-project.org/web/packages/ggvenn/ggvenn.pdf (2021).

[100] Oksanen J The vegan package. Community Ecol. Package 10, 719 (2007).

[101] Thissen D, Steinberg L & Kuang D Quick and easy implementation of the Benjamini-Hochberg procedure for controlling the false positive rate in multiple comparisons. J. Educ. Behav. Stat 27, 77–83 (2002).

