# Author Correction: The potential role of early life feeding patterns in shaping the infant fecal metabolome: implications for neurodevelopmental outcomes

**DOI:** 10.1038/s44324-025-00071-4

**Published:** 2025-06-06

**Authors:** Bridget Chalifour, Elizabeth A. Holzhausen, Joseph J. Lim, Emily N. Yeo, Natalie Shen, Dean P. Jones, Bradley S. Peterson, Michael I. Goran, Donghai Liang, Tanya L. Alderete

**Affiliations:** 1 Department of Integrative Physiology, University of Colorado Boulder, Boulder, CO USA; 2 Rollins School of Public Health, Emory University, Atlanta, GA USA; 3 School of Medicine, Emory University, Atlanta, GA USA; 4 Children’s Hospital Los Angeles, Los Angeles, CA USA

**Keywords:** Physiology, Metabolomics

Correction to: *npj Metabolic Health and Disease* 10.1038/s44324-023-00001-2, published online 13 December 2023

In the original version of this Article, Author Contributions for Natalie Shen and Joseph J. Lim were missing. The original article has been corrected.

