# Supplementary material for: The potential role of early life feeding patterns in shaping the infant fecal metabolome: implications for neurodevelopmental outcomes
Source: NPJ Metab Health Dis. Author manuscript; Available in PMC 2024 Jan 31. (PMC10828959; doi:10.1038/s44324-023-00001-2)
Supplement: STable and Fig [file NIHMS1960264-supplement-STable_and_Fig.pdf]

Supplemental Table 1. Mother-infant dyads who were included in the present analysis did not differ significantly at baseline from those who were excluded due to missing data.

	Included in Analysis<br>Mean ± SD or N, %<br>N = 112	Excluded from Analysis<br>Mean ± SD or N, %<br>N = 107	P-value
**Maternal Characteristics**			
Age at visit (years)	29.0 ± 6.3	28.9 ± 6.0	0.92*
Socioeconomic status (SES)	26.5 ± 12.0	26.5 ± 12.5	0.98*
Pre-pregnancy BMI (kg/m^2)	28.5 ± 5.8	28.6 ± 6.2	0.91*
**Infant Characteristics**			
Age (days)	32.5 ± 3.4	32.7 ± 5.7	0.50*
Sex (female, male, %female)	60, 52, 53.6%	57, 50, 52.8%	0.92
Mode of Delivery (c-section, vaginal, %c-section)	31, 81, 27.7%	22, 85, 22.4%	0.53
Age of solid foods (months)	5.9 ± 1.7	5.3 ± 0.6	0.55*
Antibiotics (yes, no, %yes)	12, 100, 10.7%	8, 99, 8.2%	0.71
Birth weight (kg)	3.4 ± 0.4	3.4 ± 0.4	0.50
Birth length (cm)	50.4 ± 2.4	50.5 ± 2.4	0.84*

Supplemental Table 1. Baseline (1-month) characteristics of 219 Latino mother-infant dyads from the Southern California Mother's Milk Study, stratified on inclusion into the current analysis. For continuous variables, independent t-tests were used to test for differences between those who were included in the analysis and those who were excluded due to missing metabolomics data, unless p-value is denoted with an asterisk (*), in which case a Wilcoxon rank-sum test was used. For categorical variables, Chi-square test were used to test for differences between those who were included compared to those who were excluded. Data reported are mean and standard deviation (SD) unless otherwise noted. Abbreviations: SES = socioeconomic status, based on Hollingshead Index (range: 3-66, lower scores indicate a lower social status), BMI = body mass index.

Supplemental Figure 1. Intensity of selected confirmed metabolites that varied by 1-month infant feeding group in the HILIC chromatography column.

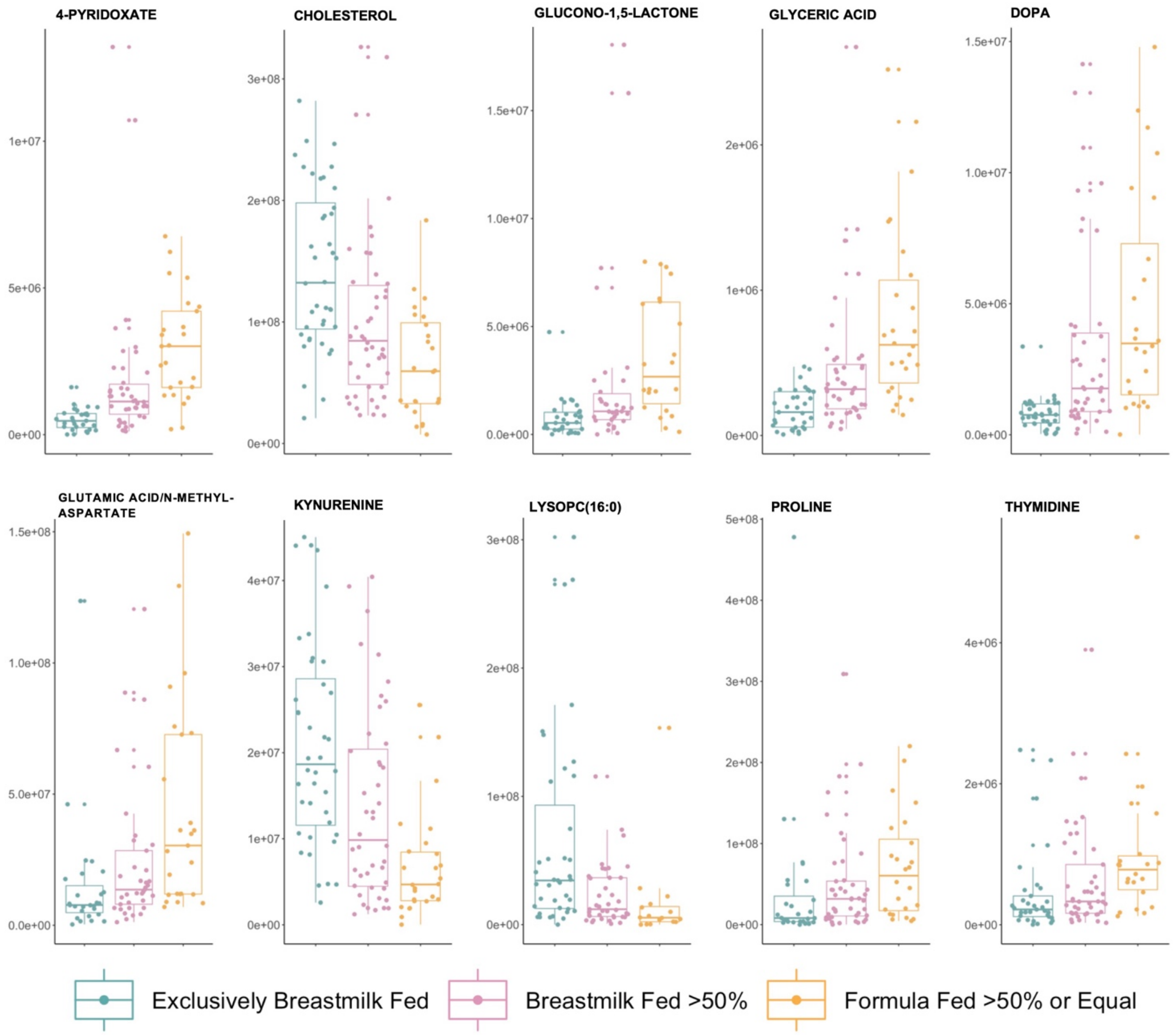

Supplemental Figure 1. Change in intensity of selected HILIC chromatography column metabolites by feeding group in infants at 1-month. Metabolites shown were the top ten that most significantly varied with infant feeding behavior. Boxplots show the median, interquartile range, and range of metabolite intensity.

Supplemental Figure 2. Intensity of selected confirmed metabolites that varied by 1-month infant feeding group in the C18 chromatography column.

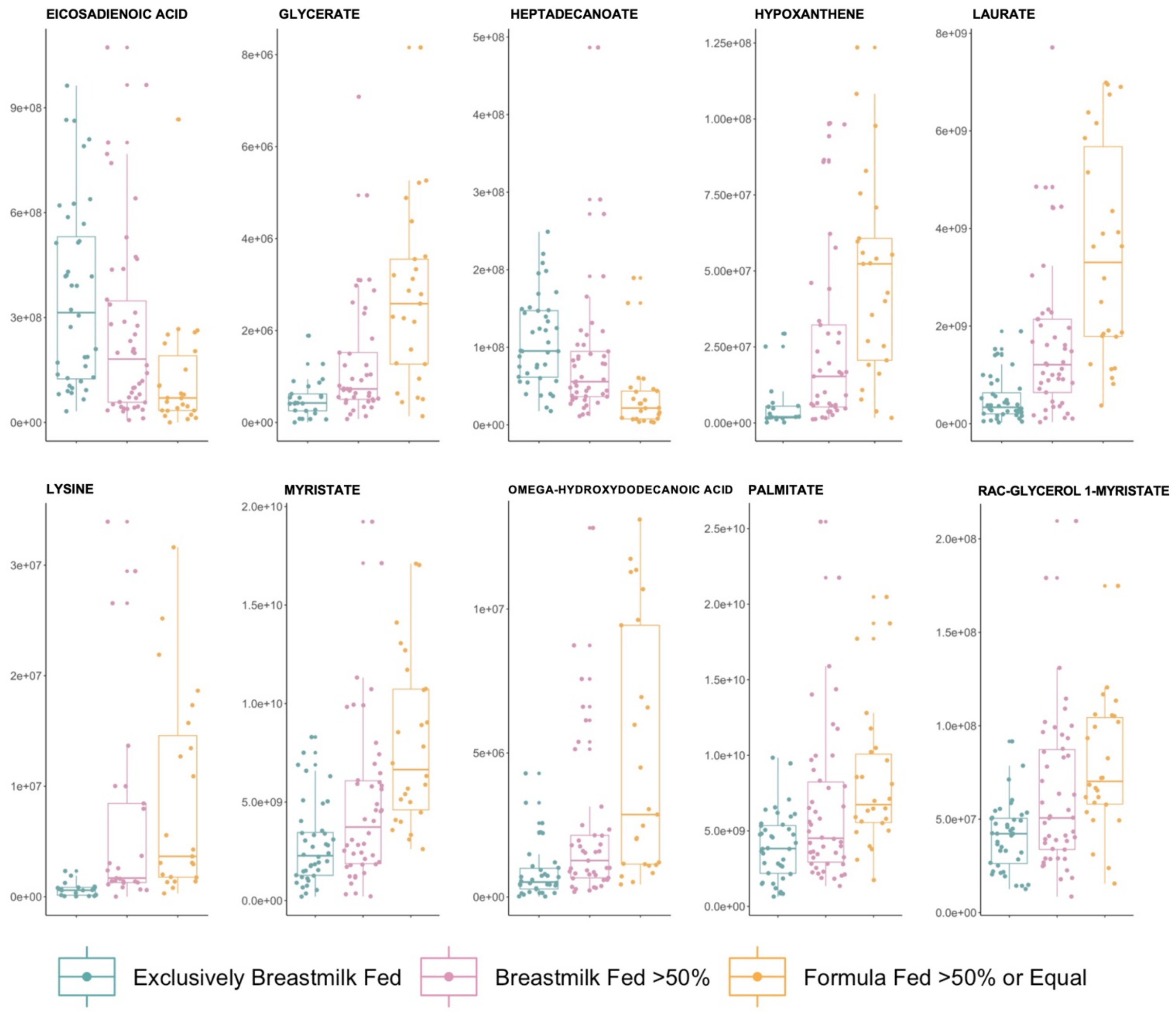

Supplemental Figure 2. Change in intensity of selected C18 chromatography column metabolites by feeding group in infants at 1-month. Metabolites shown here were the top ten that most significantly varied with infant feeding behavior. Boxplots show the median, interquartile range, and range of metabolite intensity.

Supplemental Figure 3. Intensity of selected confirmed metabolites that varied by 6-month infant feeding group in the HILIC chromatography column.

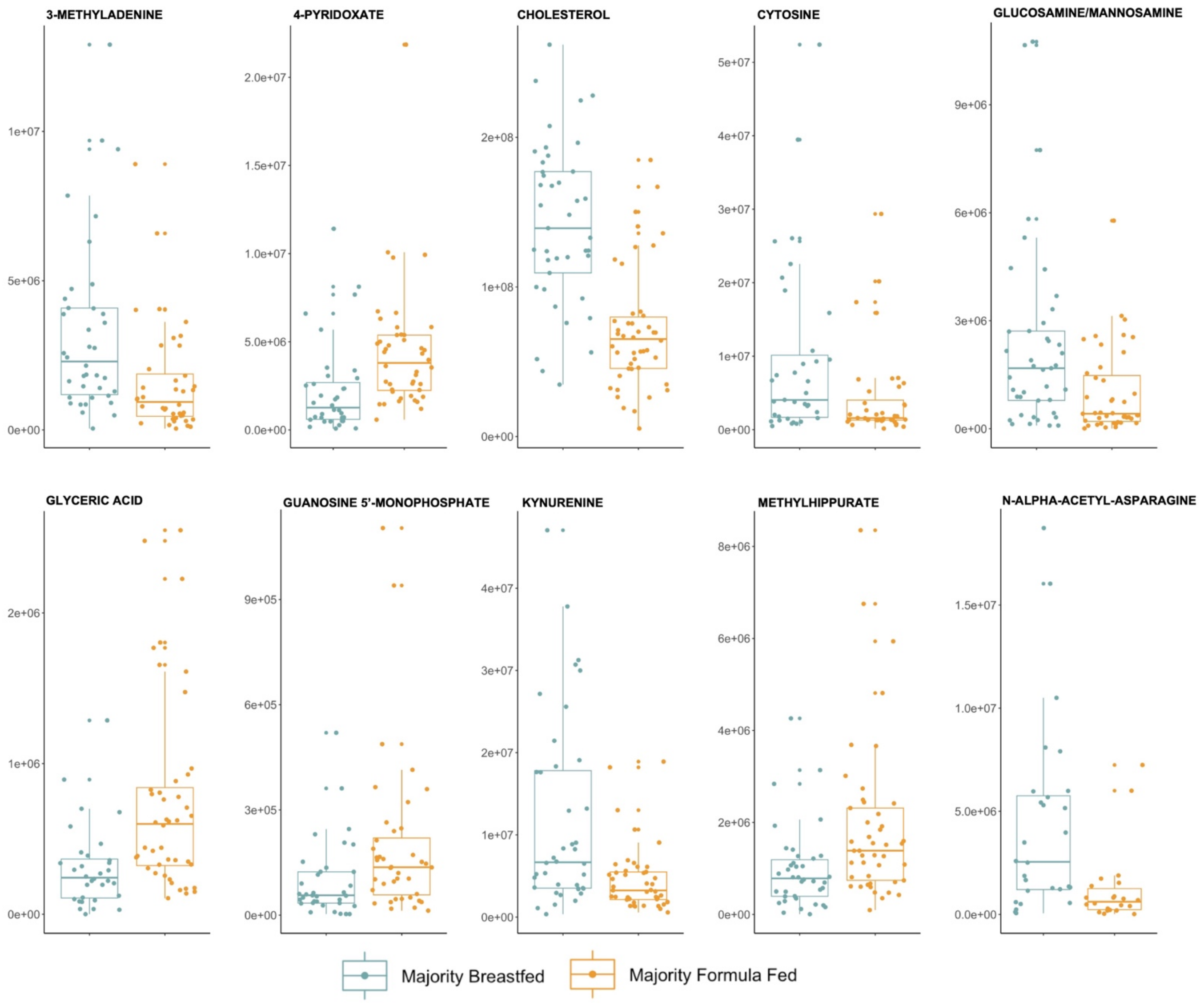

Supplemental Figure 3. Change in intensity of selected HILIC chromatography column metabolites by feeding group at 6-months. Feeding groups all include complementary solid food in the infant diet, labels represent if solid food was complimented with breastmilk feedings as a majority, or formula feedings as a majority. Metabolites shown here were the top ten most significantly varied with infant feeding behavior. Boxplots show the median, interquartile range, and range of metabolite intensity.

Supplemental Figure 4. Intensity of selected confirmed metabolites that varied by 6-month infant feeding group in the C18 chromatography column.

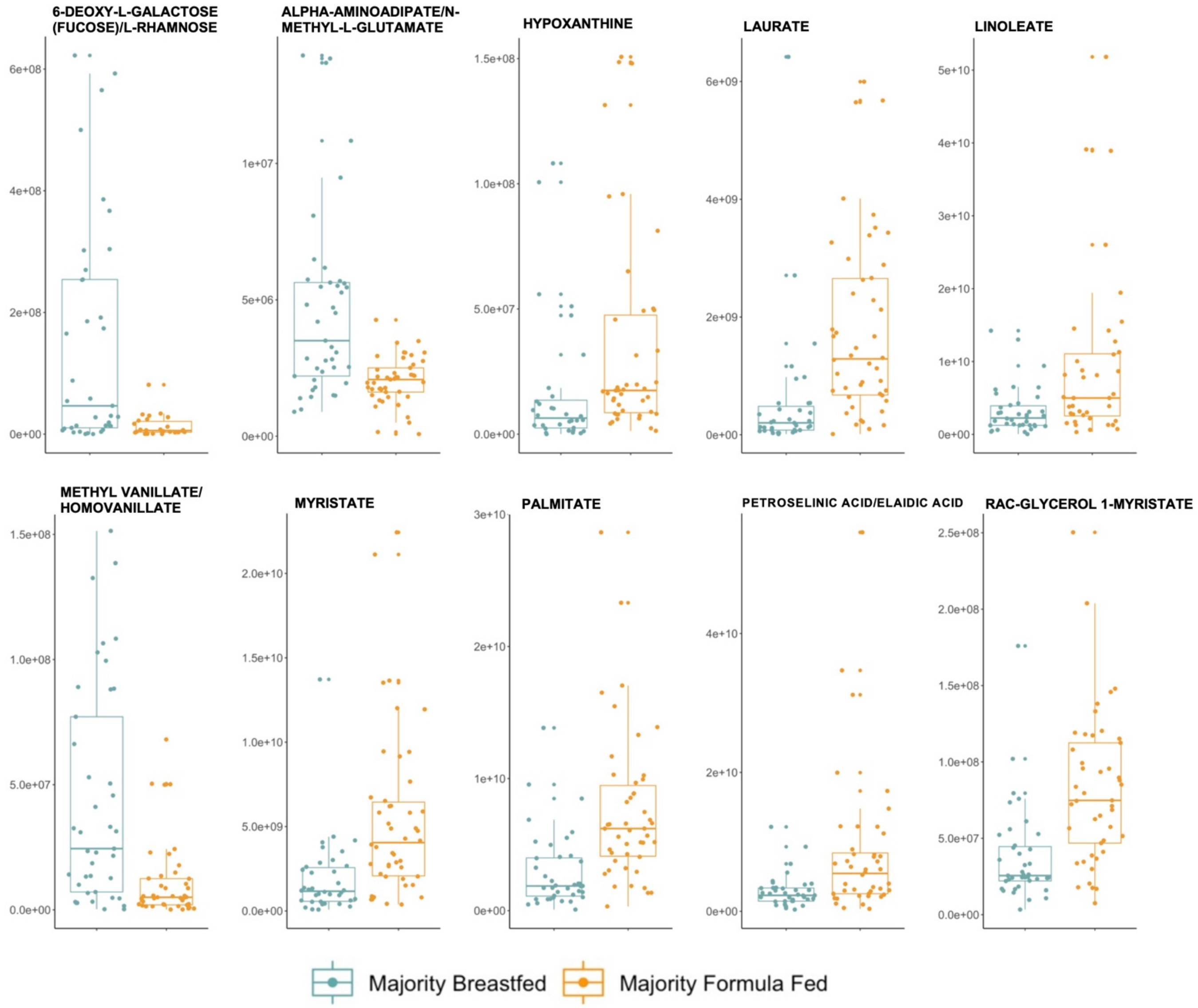

Supplemental Figure 4. Change in intensity of selected C18 chromatography column metabolites by feeding group at 6-months. Feeding groups all include complementary solid food in the infant diet, labels represent if solid food was complimented with breastmilk feedings as a majority, or formula feedings as a majority. Metabolites shown here were the top ten that most significantly varied with infant feeding behavior. Boxplots show the median, interquartile range, and range of metabolite intensity.

Supplemental Figure 5. Associations between 1-month HILIC metabolites and Bayley Scores at 2 years.

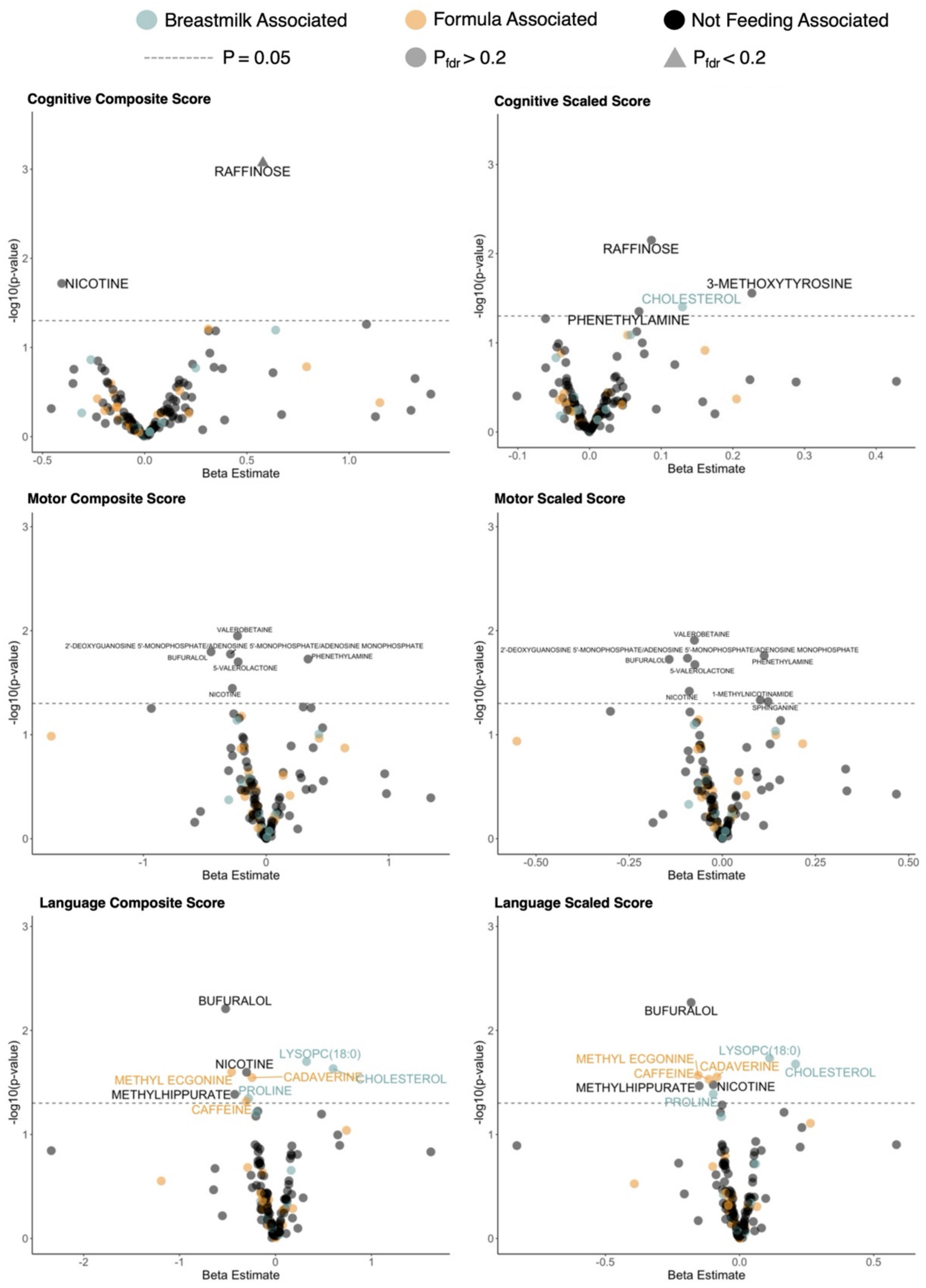

Supplemental Figure 5. Estimates were generated using linear models that adjusted for infant birth weight and mode of delivery. P-values were adjusted for multiple testing using the Benjamini-Hochberg (BH) procedure. The dashed grey line corresponds to P=0.05. Points are colored by previous feeding association and triangular points indicate $P_{BH}<0.2$.

Supplemental Figure 6. Associations between 1-month C18 metabolites and Bayley Scores at 2 years.

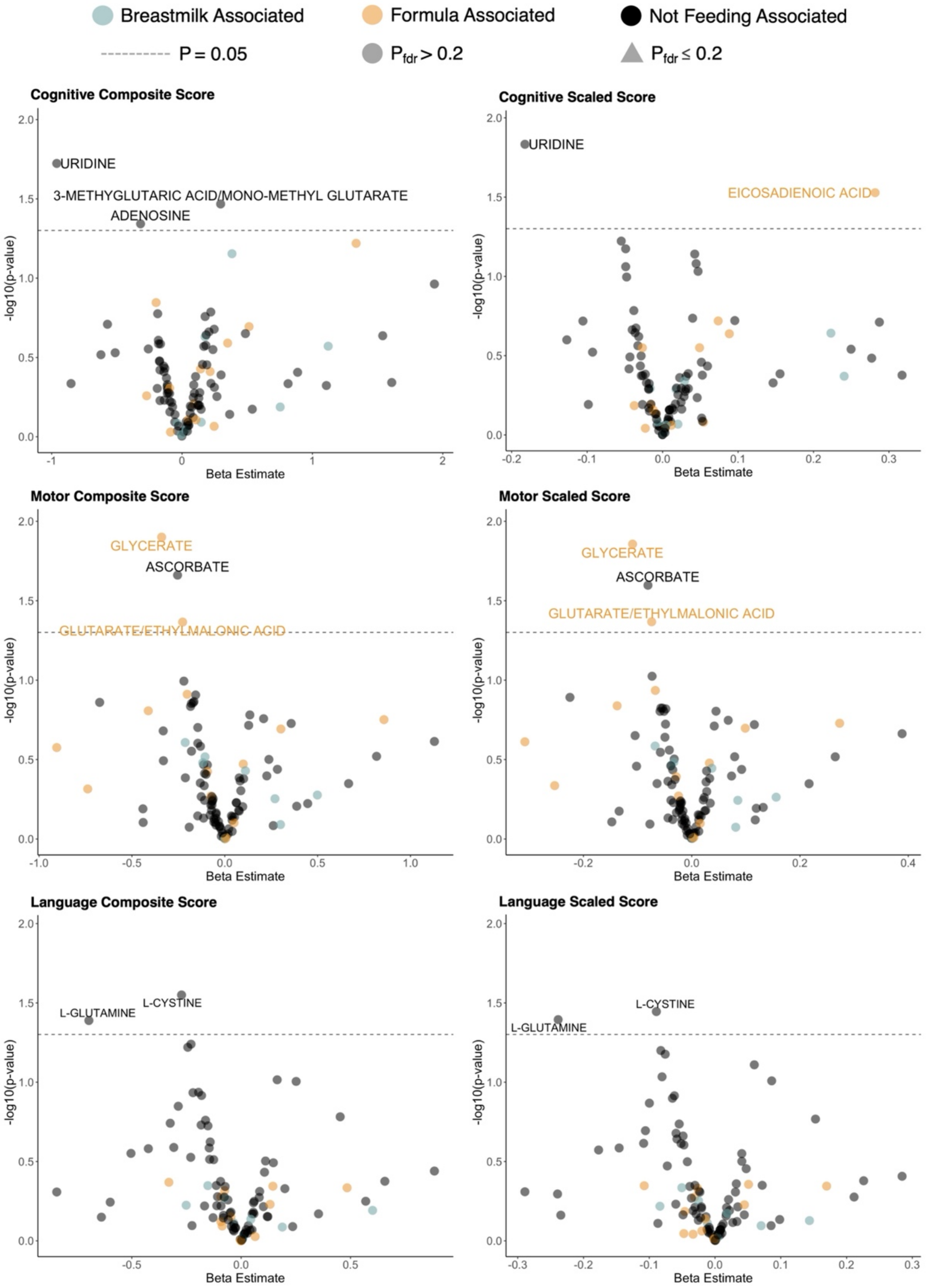

Supplemental Figure 6. Estimates were generated using linear models that adjusted for infant birth weight and mode of delivery. P-values were adjusted for multiple testing using the Benjamini-Hochberg (BH) procedure. The dashed grey line corresponds to P=0.05. Points are colored by previous feeding association and triangular points indicate P_{BH}<0.2.

Supplemental Figure 7. Associations between 6-month HILIC metabolites and Bayley Scores at 2 years.

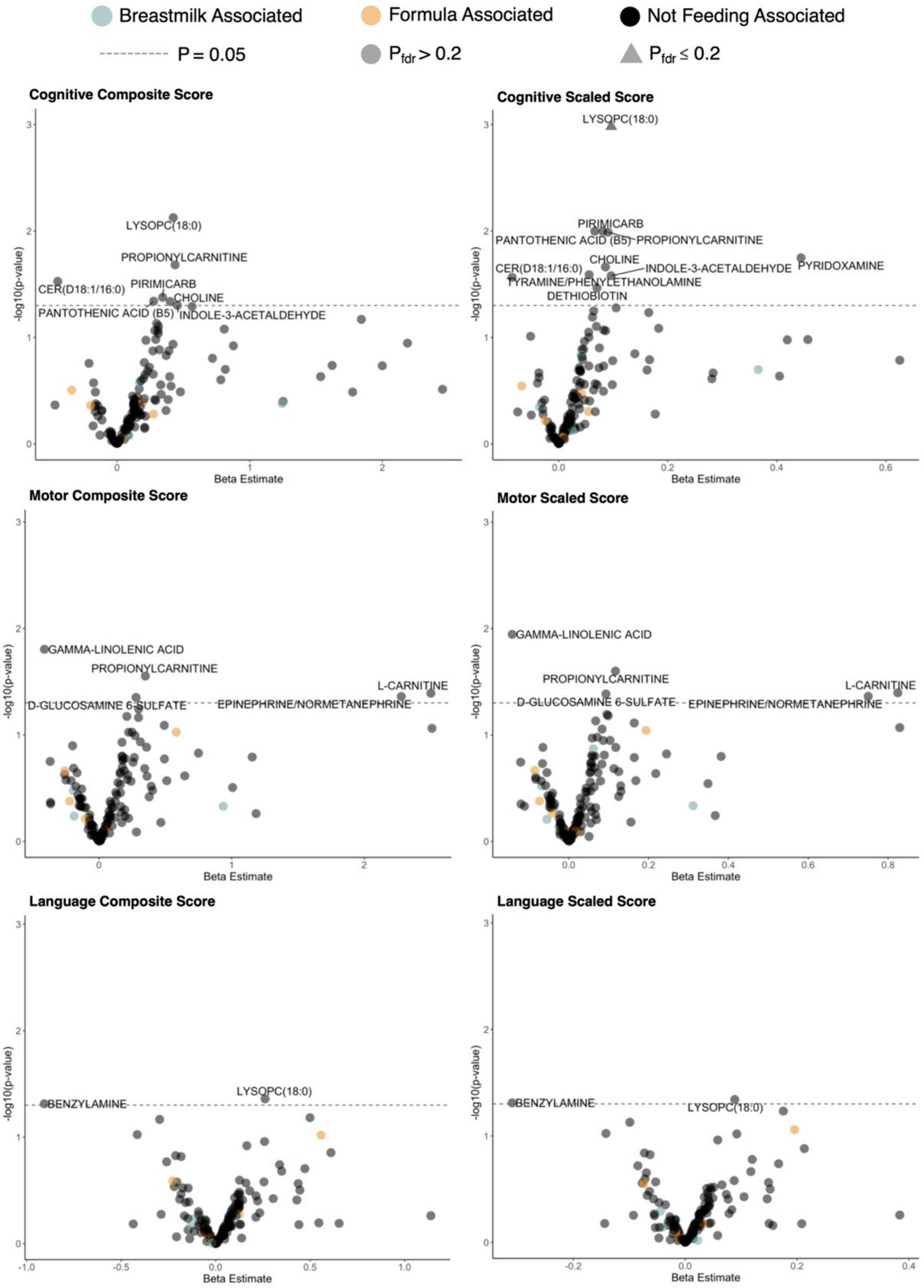

Supplemental Figure 7. Estimates were generated using linear models that adjusted for infant birth weight and mode of delivery. P-values were adjusted for multiple testing using the Benjamini-Hochberg (BH) procedure. The dashed grey line corresponds to P=0.05. Points are colored by previous feeding association and triangular points indicate $P_{BH}<0.2$.

Supplemental Figure 8. Associations between 6-month C18 metabolites and Bayley Scores at 2 years.

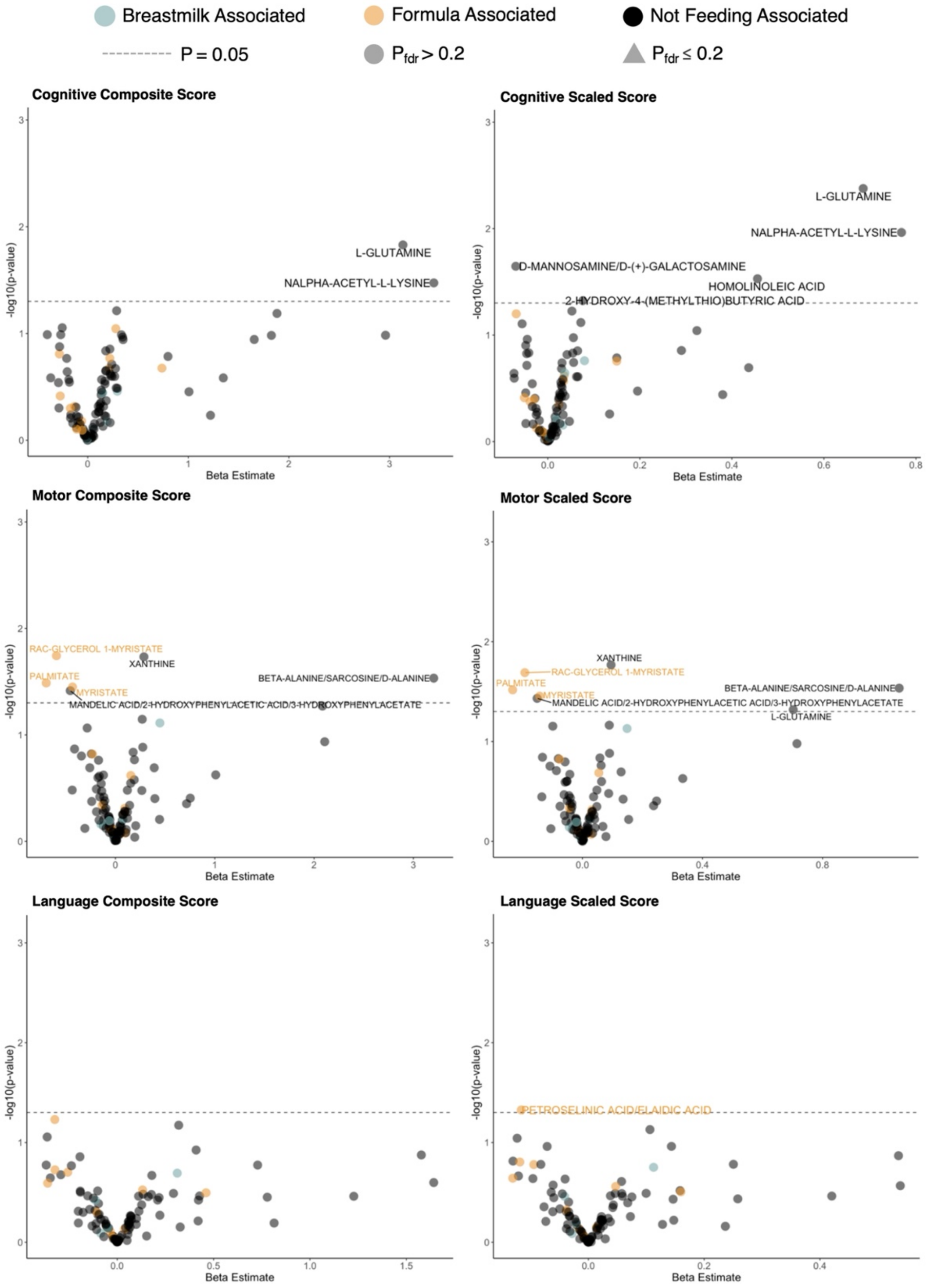

Supplemental Figure 8. Estimates were generated using linear models that adjusted for infant birth weight and mode of delivery. P-values were adjusted for multiple testing using the Benjamini-Hochberg (BH) procedure. The dashed grey line corresponds to P=0.05. Points are colored by previous feeding association and triangular points indicate $P_{BH}<0.2$.

Supplemental Figure 9. Flow chart of participants utilized in analysis.

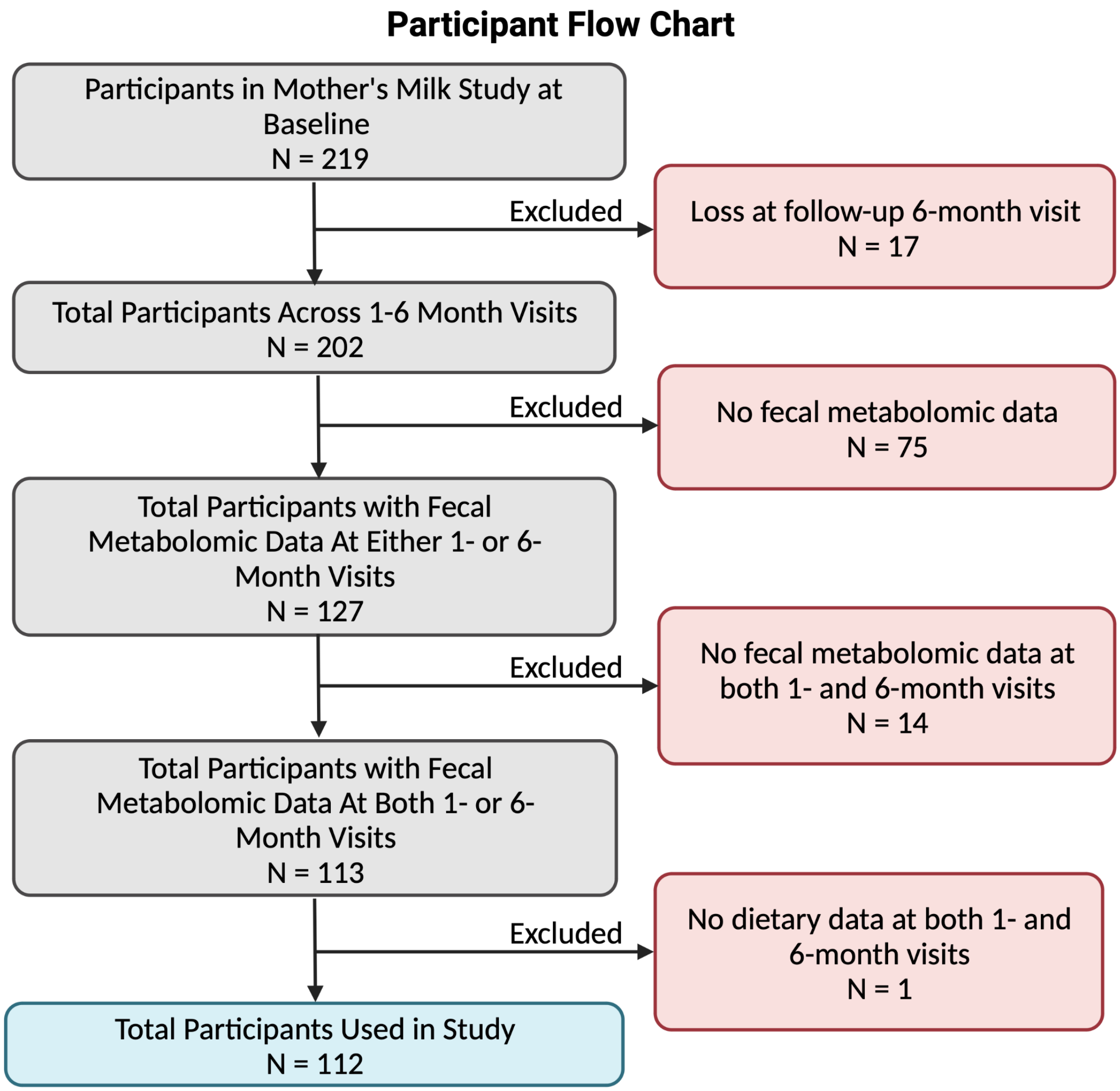

Supplemental Figure 9. Flow chart describing participant selection for inclusion in the analysis. Figure was created with BioRender.com.
